# Diverse Phases of Carbonaceous Materials from Stochastic Simulations

**DOI:** 10.1021/acsnano.0c08029

**Published:** 2021-03-15

**Authors:** Susanna Monti, Giovanni Barcaro, William A. Goddard, Alessandro Fortunelli

**Affiliations:** † ThC2-Lab and Molecular Modelling Team, CNR-ICCOM & IPCF, Consiglio Nazionale delle Ricerche, via Giuseppe Moruzzi 1, 56124 Pisa, Italy; ‡ Materials and Process Simulation Center (MSC), California Institute of Technology, Pasadena, California 91125, United States

**Keywords:** PES transformations, global optimization, graphitic phases, pore-size distribution, amorphous carbon, glassy carbon, carbon nanotubes

## Abstract

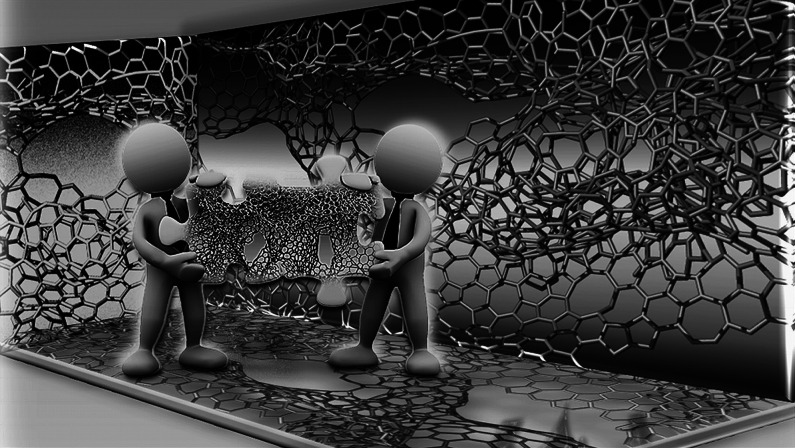

Amorphous carbon systems are emerging to have unparalleled properties at multiple length scales, making them the preferred choice for creating advanced materials in many sectors, but the lack of long-range order makes it difficult to establish structure/property relationships. We propose an original computational approach to predict the morphology of carbonaceous materials for arbitrary densities that we apply here to graphitic phases at low densities from 1.15 to 0.16 g/cm^3^, including glassy carbon. This approach, dynamic reactive massaging of the potential energy surface (DynReaxMas), uses the ReaxFF reactive force field in a simulation protocol that combines potential energy surface (PES) transformations with global optimization within a multidescriptor representation. DynReaxMas enables the simulation of materials synthesis at temperatures close to experiment to correctly capture the interplay of activated *vs* entropic processes and the resulting phase morphology. We then show that DynReaxMas efficiently and semiautomatically produces atomistic configurations that span wide relevant regions of the PES at modest computational costs. Indeed, we find a variety of distinct phases at the same density, and we illustrate the evolution of competing phases as a function of density ranging from uniform *vs* bimodal distributions of pore sizes at higher and intermediate density (1.15 g/cm^3^ and 0.50 g/cm^3^) to agglomerated *vs* sparse morphologies, further partitioned into boxed *vs* hollow fibrillar morphologies, at lower density (0.16 g/cm^3^). Our observations of diverse phases at the same density agree with experiment. Some of our identified phases provide descriptors consistent with available experimental data on local density, pore sizes, and HRTEM images, showing that DynReaxMas provides a systematic classification of the complex field of amorphous carbonaceous materials that can provide 3D structures to interpret experimental observations.

Carbon-based materials exhibit unique physicochemical properties that combine such desirable characteristics as high mechanical strength, high electrical and thermal conductivity, and diverse and flexible chemical bonding, making them appropriate for many applications, connected to hard coatings,^1,2^ electrodes,^3^ supercapacitors,^4−7^ sensors,^8^ optical materials,^9,10^ deposition substrates for catalysts,^11−15^ and catalytic materials *per se*.^16−18^ In addition to ordered phases (graphite and diamond) and bulk^19^ and low-dimensional structures (nanotubes,^20^ graphene,^21^ and nanodiamond,^22^*etc.*), amorphous carbon systems provide unparalleled properties at multiple length scales, making them the preferred choice for creating advanced materials in many sectors.^3,13,23,24^ However, the lack of long-range order has made it difficult to characterize these carbonaceous materials microscopically so that their structure/property relationships remain uncertain. Early experimental studies singled out the mass density as a fundamental descriptor in the carbon phase diagram, showing how conductivity, mechanical strength, and electron energy loss spectra (EELS) all correlate with density.^25^ More recently, the quest for systems having improved functional performance has led to the development of a variety of diverse phases, including *distinct materials at the same density*. This demonstrates that descriptors other than simple mass density must be defined to fully characterize these carbon materials. Despite experimental advances, the detailed atomistic structure of these glassy and amorphous phases remains a subject of debate.^26^ Atomistic simulations have been employed, in synergy with experiments, to improve interpretation of the data, to provide plausible microscopic configurations for the materials, and to uncover the mechanisms responsible for the formation of complex structures.^27−29^ However, such computational studies face severe challenges in identifying representative structures in a rugged potential energy landscape,^30,31^ characterized by a multitude of local minima separated by high anisotropic barriers connected with breaking and formation of strongly covalent directional bonds. Typically, these issues have been tackled using simulated annealing molecular dynamics (MD) simulations in which the system is heated to high temperatures to overcome these barriers and then cooled to find nearby minimum energy structures. Advanced protocols have also been designed to simulate dynamical processes of the carbonaceous materials comprising the formation and growth of complex structures.^32−36^ Despite the insights achieved,^2,27,28,32−42^ MD techniques suffer from limitations in the parameters that can be tuned to fully scan the structural degrees of freedom in practical simulation times,^37,41,42^ often exploring only a limited part of the phase space of these diverse systems. Another issue is that high temperatures, at the verge of melting (≈4000 K^38,39^), are generally used in the MD simulated annealing stage to accelerate atomic rearrangements (temperatures often much higher than those used in experiment, ≈2000 K, to obtain phases dominated by graphitic character^43^ while simultaneously allowing well-defined pore structure that is crucial for many applications^44,45^). An alternative approach, based on an inverse scheme, in which atomistic models are derived from a search over a space of configurations matching specific experimental quantities, has produced encouraging results,^46−48^ but its validity in terms of exhaustive characterization has been criticized.^46,48,49^ In general, available experimental input has proved insufficient to identify all of the important descriptors needed to discriminate among regions of the enormous phase space of these systems. The above considerations suggest that obtaining an in-depth understanding of the morphology of amorphous carbonaceous materials requires a systematic classification of their potential energy surfaces (PES) based on simulation protocols working at conditions (*e.g.*, temperature) comparable with real experiments.

To overcome this impasse to produce the thorough structural descriptions needed for interpreting the complex structures in this field, we explore, develop, and test an alternative approach, the dynamic reactive massaging of the potential energy surface (DynReaxMas). Our strategy is based on reactive force-field (ReaxFF) modeling^32,50^ that we use for an original set of simulations combining PES transformations^51^ and global optimization (GO) searches^52^ within a multidescriptor representation (Figure 2.9 in ref (53)). As reported below, our protocol can identify efficiently and semiautomatically candidate atomistic configurations that span the relevant regions of the PES at an affordable computational cost. This is demonstrated by its ability to find quickly different phases *at the same density*. Moreover, we can conduct simulations at a range of synthesis temperatures, including the one used in experiment (≈2000 K). This demonstrates how important it is to work at realistic conditions to obtain the proper morphology of phases (as opposed to annealing at very high temperatures).^43−45^ Indeed, we show that some of the identified phases, not found within previous simulations, provide descriptors consistent with available experimental data, depicting realistic scenarios with interpretations of experimental observations.^41,54−58^ Finally, these results suggest that our strategy offers a promising tool for investigating the introduction of other elements (O, N, and P, *etc.*, dopants) into the carbonaceous materials matrices.

This work is organized as follows. We first describe the DynReaxMas approach, providing the theoretical justification and detailing its application specifically to graphitic carbonaceous materials. We then implement DynReaxMas, focusing attention on three distinct representative densities, showing how diverse phases at the same density can be produced by our algorithm, and cataloging them in terms of descriptors. We also validate and benchmark the method by comparing with experimental data. Finally, we summarize our main conclusions.

## Results and Discussion

We applied the DynReaxMas simulation protocols to several models, ranging in size from 4176 to 25056 carbon atoms (50112-atom models were also explored). In the following, we will discuss the most significant results for the models containing 25056 carbon atoms obtained while changing the massaging parameters according to the schemes prototyped in eqs 1, 2, and 3, *i.e.*, the MM1/MM8, MM2/MM6, and MM3/MM4 FFM massages. We will also mention results from the nine possible combinations MMi/MMj (with *i* = 1, 2, 3; *j* = 4, 6, 8) = {MM1, MM2, MM3} × {MM4, MM6, MM8} obtained using all combinations of FFM massages as discussed in Theoretical Approach and Justification and reported in full in the Supporting Information (SI). Note that we put a major focus on *medium-length-scale features of the material* (1–4 nm), which are less investigated, also aiming at triggering development of further experimental characterization tools and analysis. From this point of view, a 25056-atom set is a balanced size: it is more realistic than 4176 atoms but computationally less demanding than, *e.g.*, the 50112-atom system. As we will see below, the 25056-atom case is large enough to investigate medium-range morphologies, such as the formation of pores and tunnels (see below bimodal *vs* homogeneous pore-size distributions also in connection with experimental small mesopores of 2–5 nm), while the MD (CPU) times needed to overcome barriers, produce phase transformations, and equilibrate the system (*e.g.*, reconstruct after disaggregation) at this size are affordable with our computational resources (further computational information on simulation times is given in the SI). As a rule, the lower the density and the bigger the number of atoms, the longer are the simulation times needed to achieve equilibration. The 4176-atom model was mainly used as a prototype to tune the methodology and to design an efficient simulation scheme.

Along with previous literature,^25^ we keep the simulated mass density as the primary descriptor and focus on three values: 0.16, 0.50, and 1.15 g/cm^3^. These values of density are in the range for graphitizable carbon^59^ and close to the experimental densities relevant for systems critical in applications such as glassy carbon electrodes and carbonaceous deposition supports (for comparison, the mass densities of diamond and graphite are 3.51 and 2.26 g/cm^3^, respectively). In the following, we will concentrate on representative configurations and their analysis in terms of descriptors at these three mass densities, with the aims of (i) highlighting the diversity of phases produced, (ii) identifying the complementary descriptor most appropriate to distinguish the diverse phases at each value of the mass density, and (iii) determining the minimum set of computational simulations needed to generate this diversity.

**Figure 1:**
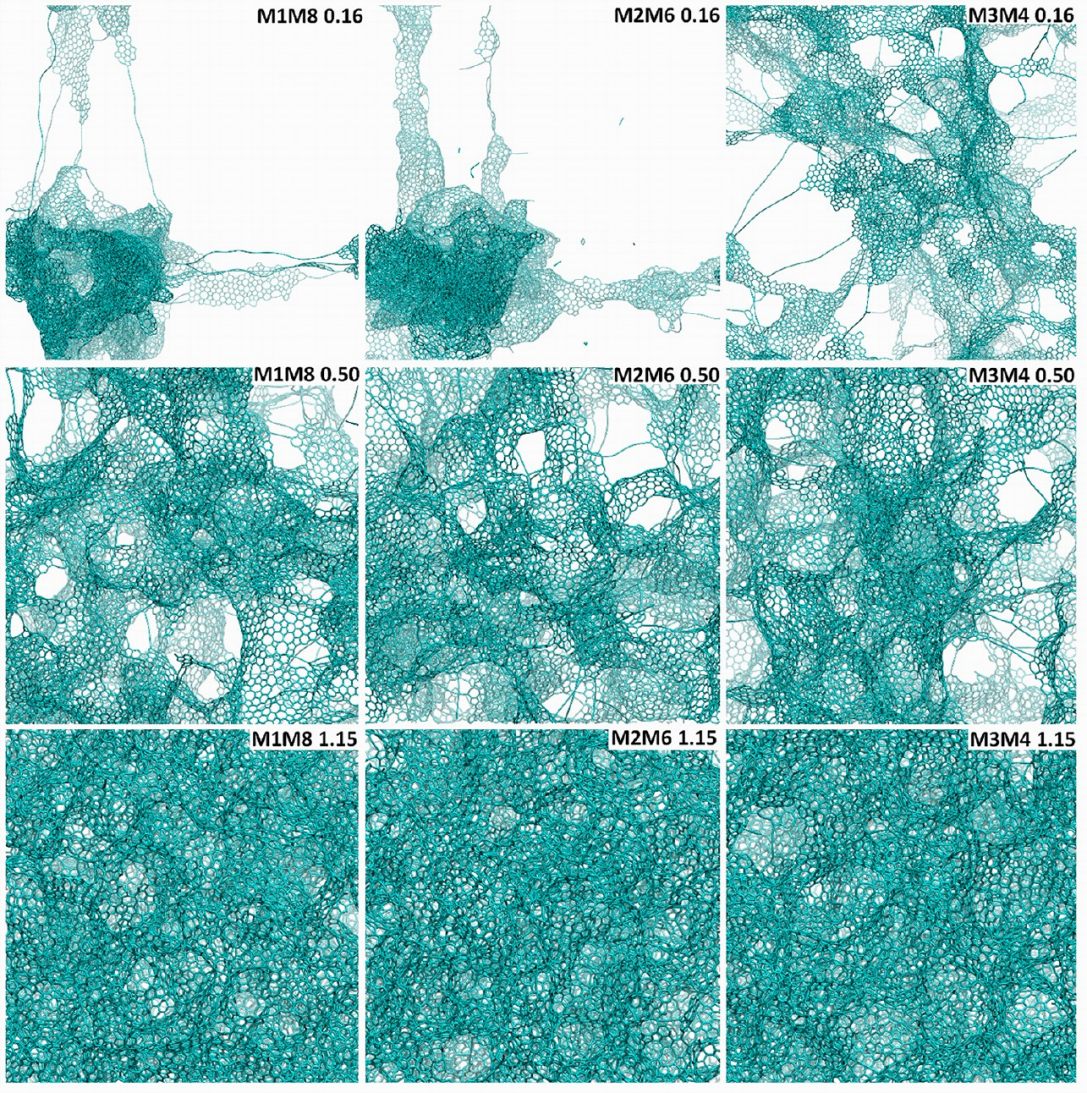
Schematic atomistic depictions of the different phases generated by the FFM prototyped massages, *i.e.*, MM1/MM8, MM2/MM6, and MM3/MM4, eqs 1–3, and selected as representative, obtained at mass densities of 0.16 (top row), 0.50 (middle row), and 1.15 g/cm^3^ (bottom row), respectively. For the sake of notation, MM1/MM4 is simplified to M1M4 in the inset and analogously for the other combinations.

Figure 1 shows schematic pictures of the phases generated by the FFM massages prototyped in eqs 1, 2, and 3, and selected as representative, at the three mass densities of 0.16, 0.50, and 1.15 g/cm^3^, respectively.

### Mass Density 0.50 g/cm^3^

For reasons that will be clear below, we start with a mass density of 0.50 g/cm^3^, intermediate among the three densities considered and most common in applications related to carbonaceous materials.

**Figure 2:**
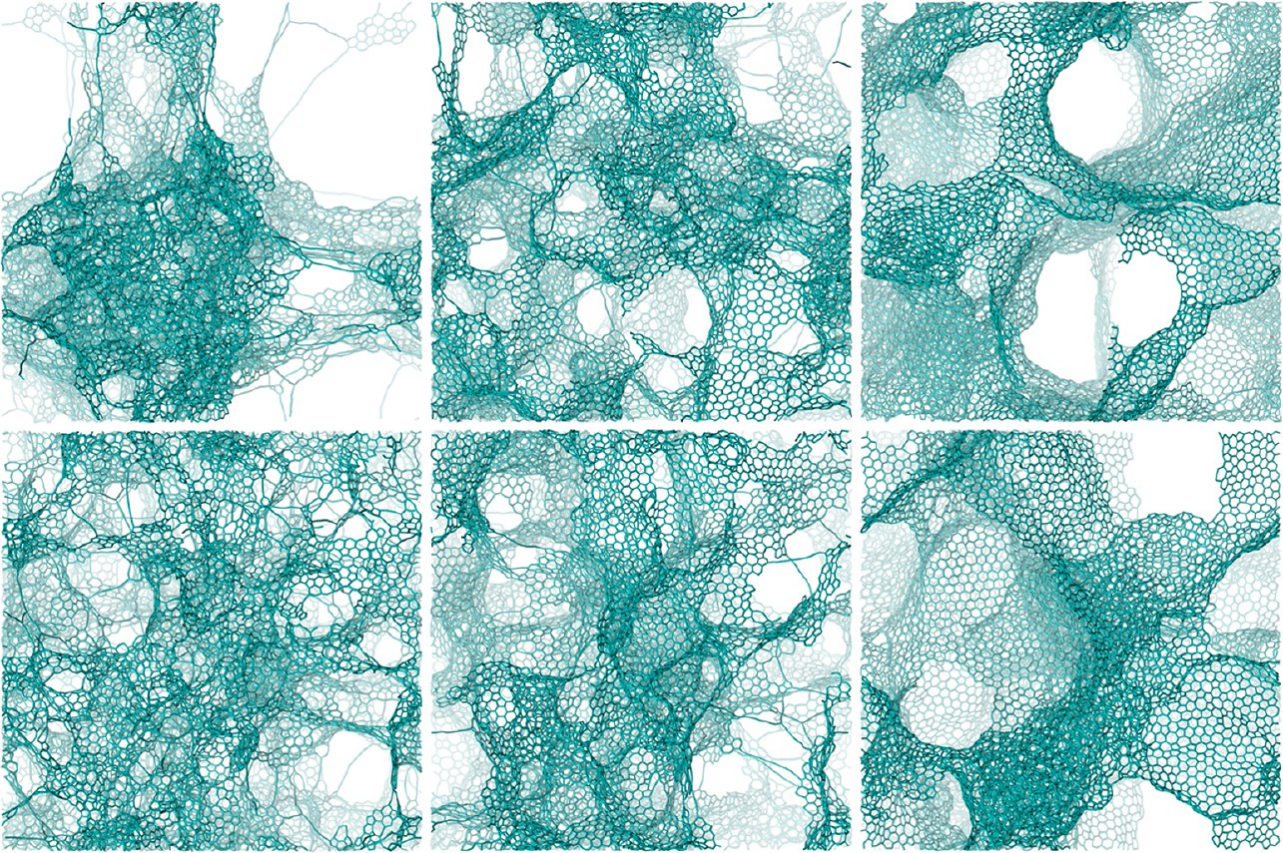
Schematic atomistic depictions of the phases generated by the MM1/MM8 (top row) and MM3/MM4 (bottom row) FFM massages, *i.e.*, eqs 1 and 2, as a function of the simulation temperature: 1500 K (left column), 2000 K (middle column), and 3000 K (right column), respectively, at a mass density of 0.50 g/cm^3^.

At this density we also performed simulations as a function of the simulated synthesis temperature, whose results are reported in full in SI Figures S7, S13, S17, and S21, and in the atomistic movies as a function of time in the final reconstructive stage (Videos S1, S2, S3, S4, S5, and S6) of the SI. In Figure 2 we condense selected information from Figure S7, depicting atomistic structures of the phases generated by the MM1/MM8 and MM3/MM4 FFM massages, eqs 1 and 2, respectively, at three different simulation temperatures: 1500, 2000, and 3000 K, respectively. These structures show pictorially how the temperature plays a crucial role in the synthesis process: the contrast between the simulations at lower temperatures on the one hand (1500 and 2000 K) and at the higher temperature on the other hand (3000 K) is visually striking. It is immediately apparent that at 1500 and 2000 K one finds a diversity of the morphologies, characterized by

(i) diverse pore-size distributions with complex pore structures (also see Figure S13);

(ii) a variable thickness (number of layers) of the pore walls (signatures in the *g*(*r*) of Figure S21);

(iii) agglomerated (see MM1/MM8 at 1500 K) *vs* sparser morphologies (where the latter can be further distinguished into boxed *vs* fibrillar motifs; more below).

This diversity is drastically simplified in the “lean” phases produced at 3000 K. For point ii, notice how the first peak of the *g*(*r*) in Figure S21 decreases at 3000 K as the morphology evolves toward individual graphitic sheets. This analysis is confirmed by the simulated HRTEM images of Figure S17, making it experimentally verifiable as discussed below at the end of this section. Particularly suggestive in this connection are the atomistic movies as a function of time of the final reconstructive stage reported in the SI (Videos S1, S2, and S3, *etc.*), which depict the evolution of the system during reconstruction. Incidentally, these movies furnish suggestions and insight (for both theory and experiment) toward arriving at general principles for the synthesis process. The picture resulting from these DynReaxMas simulations is perfectly consistent with the empirical finding that annealing at ≈2000 K (around 1800–2000 °C) is needed experimentally to obtain highly conductive amorphous carbonaceous materials^43^ but also that a well-defined pore structure which is crucial for applications^44,45^ is progressively lost when synthesis is conducted at too high temperatures, especially beyond the full graphitization temperature of 2550 K.^59^ Our work thus provides microscopic insight and support to the empirical search of synthesis protocols working at lower temperatures to attain complex graphitic phases.

Focusing then on the prototyped DynReaxMas simulations at 2000 K, Figure 1 (middle) shows the three phases obtained at a mass density of 0.50 g/cm^3^*via* the *massaging* parameters defined in eqs 1, 2, and 3.

Here we employ descriptors to analyze and catalog results and to identify and classify the phases. For each structure, we evaluate all of the defined descriptors and distinguish two configurations as qualitatively different when the mismatch of at least one of these descriptors exceeds a given threshold or by visual inspection in the case of descriptors given as functions, such as the plots of pore-size distribution or the HRTEM images. As anticipated above, we find that the given combinations of FFM massages produce various levels of graphitization (in terms of condensed ring systems), and different pore structures with a variable thickness (number of layers) of the walls, and different morphologies (agglomerated *vs* sparser and into boxed *vs* fibrillar).

**Figure 3:**
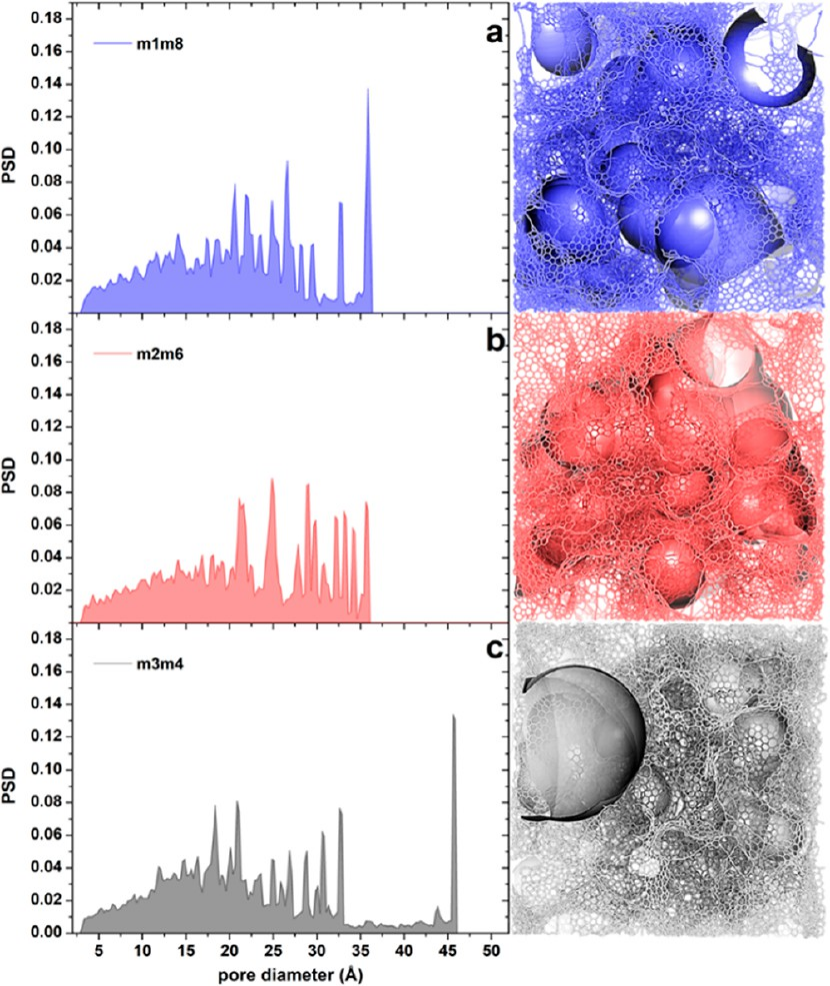
Schematic depiction of the pores in terms of the pore-size distribution (PSD, left panels) and pictorial illustrations (right panels) for the different phases generated by the FFM prototyped massages, *i.e.*, MM1/MM8 (top row), MM2/MM6 (middle row), and MM3/MM4 (bottom row), eqs 1–3, and selected as representative, obtained at a mass density of 0.50 g/cm^3^, respectively. Pores are identified, and PSD plots are produced, *via* the Poreblazer software,^74^ while real-space pictures in the right panels are produced using the CAVER software^75^ and a variable probe radius. Noteworthy is the competition between a more homogeneous MM1/MM8 (top row), MM2/MM6 (middle row), or bimodal MM3/MM4 (bottom row) distribution of pore sizes. For the sake of notation, MM1/MM8 is simplified to M1M8 in the inset and analogously for the other combinations.

In particular, at a mass density of 0.50 g/cm^3^ in the targeted (graphitic) region of the PES, we found that the distribution of pore sizes provides a proper complementary descriptor to differentiate phases, as confirmed by the PSD of Figure 3. Pores are identified *via* the Poreblazer software,^74^ while real-space pictures in Figure 3 (and other graphics) have been produced using the CAVER software,^75^ which uses spherical probes to fill void regions of the structures: by varying the radius of the probe, we can estimate the range of probe radii contained in any given pore. In the left panels of Figure 3, pores are visualized by scanning the probe radius starting from the largest value and then decreasing the probe radius, limiting collection to the first 10–15 pores, so that exceedingly small pores are not considered significant and not reported.

Using this analysis tool, at the mass density of 0.50 g/cm^3^ we find a competition between homogeneous (strikingly MM2/MM6, but also MM1/MM8) and heterogeneous (MM3/MM4) structures, *i.e.*, structures with a more uniform (MM2/MM6, MM1/MM8) or bimodal (MM3/MM4) distribution of the pore sizes. The bimodal case, with the coexistence of large and small sizes, is illustrated quantitatively in Figure 3 and is consistent with experiments.^56^ Due to limitations in the system size and in statistics, at this stage we cannot state whether there is a continuum of phases going from homogeneous to bimodal or the two morphology classes are discontinuously separated—we only claim that these are distinct phases and experimentally realized.

Using the CAVER code, which can identify “tunnels” in addition to pores, *i.e.*, extended and connected portions of void spaces, we could observe that the inhomogeneous distribution of pore sizes also leads to very narrow tunnels simultaneously with large ones. A representative narrow tunnel is shown in Figure S22. In contrast, in the homogeneous phases, tunnels and pores are more uniform and generally larger. These differences may be useful for some applications. Mass transport of even relatively big molecules should be facile in the large tunnels of the MM3/MM4 phase,^56^ while its aggregated regions may provide anchoring sites with peculiar features. Thus, it may be possible to selectively support species or functional groups potentially distinct from those typical of more extended graphitic leaflets. In contrast, the MM2/MM6 and MM1/MM8 phases may be of interest for applications needing a uniform distribution of active sites with homogeneous transport and access of small molecules to such sites.

In Table 1 we report a selection (from SI Tables S4–S6) of the most significant descriptors for the phases generated by the prototyped FFM massages at the three mass densities investigated here. In general, all phases are dominated by C_3_ atoms, accounting for about 90% of the total as targeted, giving rise to 14–16% of six-membered rings (perfect graphite would give 33%) and to 9 and 10% each of five-membered and seven-membered rings (roughly simplifying, we can say that half of the rings in the graphitic leaflets are six-membered, while the other half are distributed between five-membered and seven-membered). Although the variations in the values of the descriptors are not huge, some interesting considerations can be drawn. For example, the small values of the angle *A*_(sp^2^)_ indicate a high quality of the phases in terms of graphitic structures, whereas larger deviations are found for *A*_(sp)_, suggesting that a fraction of the C_2_ atoms are not sp-hybridized atoms but are in fact undercoordinated sp^2^-hybridized. By extracting the bond order of each atom as defined by ReaxFF, we can further quantify this indication and find that about 20–35% of the C_2_ atoms are undercoordinated sp^2^-hybridized. These atoms are the most natural candidate sites for passivation with hydrogen or simple oxygenated groups (OH, COOH, and so on) or more complex oxygenated, nitrogenated, and so on residues acting as anchoring points for catalytic or sensing functionalities.^12−15^ Passing to medium-range descriptors, at this mass density of 0.50 g/cm^3^ a clear difference is obtained by analyzing the correlation between PLD and LCD values: in the case of the homogeneous M2M6 phase. In fact, we find the largest value of PLD together with the smallest value of LCD, which confirms a major degree of uniformity in contrast with the M3M4 phase, for which we find coexistence of the smallest value of PLD and one of the largest LCD.

**Table 1:**
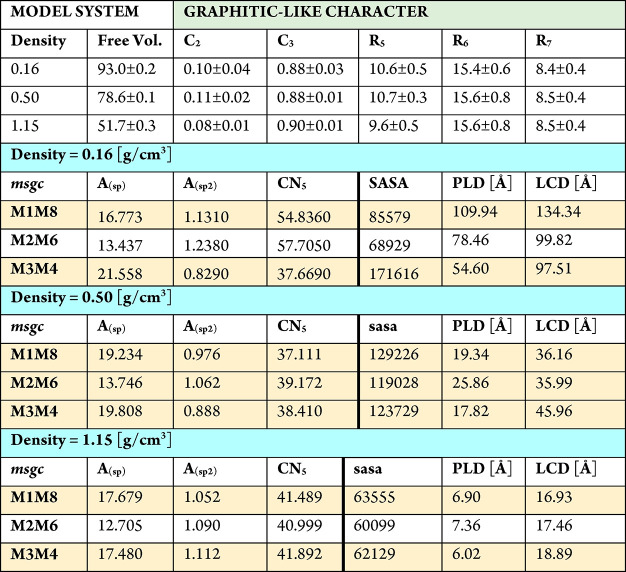
Selection of Structural Descriptors of the Final Geometries Generated by the Full DynReaxMas Protocol (Massages, Equilibration, and GO) Using the Three Prototyped DynReaxMas Massages, Namely, MM1/MM8, MM2/MM6, and MM3/MM4, Conducted at 2000 K and Various Mass Densities As Noted^a^

a MM1/MM8 is simplified to M1M48in the table for the sake of notation and analogously for the other combinations. *C*_2_ = fraction of second-coordination C atoms; *C*_3_ = fraction of third-coordination C atoms; *A*_(sp)_ = average difference between ideal 180° angle for sp C and second-coordination C angles (large values correspond to “bent wires” deviating from perfect linearity (degrees)); *A*_(sp^2^)_ = average difference between ideal 120° angle for sp^2^ C and third-coordination C angles (large values correspond to “bent sheets” deviating from the perfect planarity (degrees)); *R*_5_ = 100 × ratio between the number of five-membered rings and the total number of atoms; *R*_6_ = 100 × ratio between the number of six-membered rings and the total number of atoms; *R*_7_ = 100 × ratio between the number of seven-membered rings and the total number of atoms; SASA = solvent-accessible surface area (Å^2^); PLD = pore limiting diameter (Å); LCD = largest cavity diameter (Å).

Despite the noticeable morphological differences, the average energy per carbon atom is similar for these three representative configurations (−163.4 kcal/mol for MM2/MM6, −163.8 kcal/mol for MM1/MM8, and 164.0 kcal/mol for MM3/MM4, respectively). This suggests similar thermodynamic stability of these phases, ignoring entropic factors, and therefore the possibility of producing both experimentally if appropriate and dedicated synthesis protocols are devised that can overcome kinetic barriers (we find that the average energy per carbon atom correlates with other descriptors, *e.g.*, with the percent of atoms having coordination number = 1 and with the local density).

**Figure 4:**
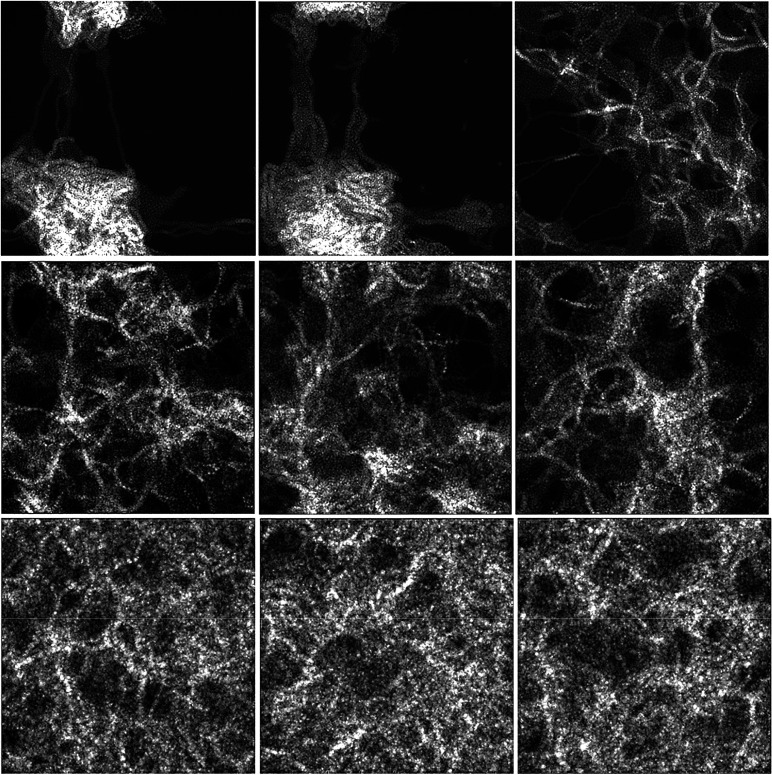
Simulated HRTEM images of the different phases obtained at a mass density of 0.16 (top row), 0.50 (middle row), and 1.15 g/cm^3^ (bottom row), generated by the FFM prototyped massages, *i.e.*, MM1/MM8 (top row), MM2/MM6 (middle row), and MM3/MM4 (bottom row), eqs 1–3, and selected as representative, respectively.

Finally, Figure 4 (middle row) shows transmission electron microscopy (TEM) images from our structural models simulated using the QSTEM software.^76,77^ A difference between the three phases is apparent (see the next paragraph and discussion at 0.16 g/cm^3^ density).

We expect these differences between the phases in pore-size distribution, HRTEM patterns, and morphology to be experimentally detectable. Indeed, the comparison with available experimental data is favorable. The literature in this field is huge, so we focus on one illustrative example. Although doubts have been recently raised on their quantitative accuracy,^84^ tools are available to extract the pore-size distribution in carbonaceous materials from an analysis of experimental data; see, *e.g.*, ref (85) for one of the most used tools. Using these tools, a few studies have identified phases exhibiting a bimodal pore-size distribution in the micro-mesopore range. For example, in ref (56) carbonaceous materials with mass densities of 0.26–0.47 g/cm^3^ were obtained *via* hydrothermal carbonization of biomass and were characterized as having a bimodal porosity made up of narrow micropores (1 nm) and small mesopores (2–5 nm), corresponding to pore volumes between 4 and 64 nm^3^, in excellent correlation with the pore volumes derived from the PSD in Figure 3 (left panels): a maximum pore diameter of 36 Å corresponds to a pore volume of ≈24 nm^3^ for the MM2/MM6 phase, whereas a pore diameter of 46 Å corresponds to a pore volume of ≈51 nm^3^ for the MM3/MM4 phase. Notably, the authors of ref (56) pointed out that a bimodal pore-size distribution is a necessary prerequisite for many applications, *e.g.*, for use in high-power applications.^56^

To conclude this subsection, we anticipate that the phases identified at this intermediate 0.5 g/cm^3^ mass density have relationships with those at the other densities. As we will see below, the phases of Figure 1 (middle rows) are related to the corresponding phases (Figure 1, bottom rows) at 1.15 g/cm^3^ and with the parallel differences in pore-size distributions for that case. Analogously, the bimodal Figure 1 (middle row, right panel) configuration will evolve into the multiwall boxed configuration of Figure 1 (top row, right panel) at 0.16 g/cm^3^, whereas the more uniform Figure 1 (middle row, middle and right panels) configurations are a prodrome of the fibrillar configurations at 0.16 g/cm^3^, Figure 1 (top row, middle and right panels).

### Mass Density 1.15 g/cm^3^

The three representative structures obtained *via* the prototyped DynReaxMas massages at a mass density of 1.15 g/cm^3^ are shown in Figure 1 (bottom rows). At this higher density, we found that the massaging protocols eqs 1, 2, and 3) converge onto a similar morphology (with some differences as discussed below). We note in passing that, to explore a larger portion of the PES, we also modified the structure generation procedure from MM3/MM4 to MM3/MM4/MM1/MM8 and obtained configurations exhibiting a C_2_/C_3_ competition, *i.e.*, graphitic *vs* chain structures (unpublished work).

The value 1.15 g/cm^3^ is a transition density between the phases at a greater mass density, typical of carbonaceous hard coatings, and phases at sparser densities associated with deposition supports and/or electrochemical applications. To single out this transition region and to distinguish its phases, it is useful to analyze the descriptors in Table 1. Some descriptors are more uniform, *e.g.*, at this higher density morphologies are characterized by a dominance of C_3_ atoms and C6 ring distributions similar to the 0.50 g/cm^3^ phases. But for CN_5_ we observe values larger by about 10% with respect to the lower-density phases, indicating an increase in short-range packing, while all of the medium-range descriptors are reduced with respect to the 0.50 g/cm^3^ phase, consistent with the density increase.

Moreover, at 1.15 g/cm^3^ mass density, we also find a competition between homogeneous (MM1/MM8 but in this case the nonprototyped massage MM1/MM6 is even more uniform, with a maximum pore diameter of 16 Å) *vs* heterogeneous (MM3/MM4, with a maximum pore diameter of 19 Å) structures, with the distribution of pore volumes as the associated complementary descriptor, exhibiting more homogeneous (MM1/MM8 or MM1/MM6) or bimodal (MM3/MM4) character, as illustrated in Figure 1 and Figure S14. The homogeneous phases MM1/MM8 and MM1/MM6 correlate with the homogeneous MM1/MM8 structure at 0.50 g/cm^3^ density, while the bimodal phase MM3/MM4 correlates with the corresponding MM3/MM4 inhomogeneous structure at 0.50 g/cm^3^ density. By further comparing the structural features at 1.15 *vs* 0.50 g/cm^3^, we note that the average values of the maximum pore volumes increase with decreasing mass density: from ≈2–4 nm^3^ at 1.15 g/cm^3^ to ≈24–51 nm^3^ at 0.50 g/cm^3^, respectively. The increase in average pore volume with decreasing density also explains why it is necessary to use structural models with a sufficiently large number of atoms (25056 in our case) to explore the régime of low mass densities (below 1 g/cm^3^); otherwise it is not possible to adequately describe pore volumes as large as 24–51 nm^3^. Once more, the average energy per carbon atom is similar for these structures (−164.4 kcal/mol for MM2/MM6, 164.8 kcal/mol for MM3/MM4, and −165.2 kcal/mol for MM1/MM8, respectively).

Finally, Figure 4 (bottom row) shows simulated TEM images from our structural models. In this case, the difference among these fully graphitic phases is less striking and may not be recognized easily *via* microscopy, whereas non-fully graphitic phases such as chain-like phases should be different and distinguishable *via* HRTEM. As an illustrative example, there is a keen interest in the use of highly graphitized carbon shells as supports in electrocatalytic applications as a promising strategy to solve the problems of electron and mass transfer,^86^ and some of these phases resemble those depicted in Figure 4, such as the HAADF-STEM images of catalysts with different heteroatom content in Figure 4 of ref (86), with a quite precise correspondence of pore sizes.

### Mass Density 0.16 g/cm^3^

Figure 1 (top row) illustrates pictorially the two phases obtained at a mass density of 0.16 g/cm^3^, *via* the prototyped massaging parameters MM1/MM8, MM2/MM6, and MM3/MM4 defined in eqs 1, 2, 3, respectively.

As visually apparent from an inspection of Figure 1 (top row), at 0.16 g/cm^3^ we find a competition between agglomerated (MM1/MM8 and MM2/MM6) *vs* sparse (MM3/MM4) configurations (resembling the competition between multiwall *vs* single-wall carbon nanotubes, CNTs).

We find several descriptors in Table 1 appropriate to differentiate these phases to complement the 0.16 g/cm^3^ mass density in the graphitic region of the PES. In detail, two groups of structures can be identified: (i) those characterized by the concomitant presence of large voids and dense-packed regions (generated by the MM1 and MM2 initial destructive protocols) and (ii) those characterized by a more uniform distribution of the atoms in the cell (generated by the MM3 initial destructive protocols). The difference between the two groups is clearly shown by the integral of the pair distribution function *g*(*r*) up to 5 Å, or CN5, which can be taken as a measure of the local or short-range density. The more homogeneous MM3/MM4 phase presents significantly lower CN5 values with respect to the more aggregated multiwall MM1/MM8 and MM2/MM6 phases, in agreement with experimental measurements.^54^ In principle, the two phases could also be differentiated by the XRD pattern (the XRD peaks around 35–40° or 80° are typical of graphitic leaflets at bonding distance and should be present in the multiwall CNT structure), but for technical reasons, we were not able to demonstrate this using the usual virtual diffraction method to simulate XRD patterns or virtual XRD analysis.^87^

Consistent with this, group i is characterized by low values of SASA and large values PLD/LCD, whereas, in contrast, group ii exhibits higher values of SASA, by about a factor of 2 with respect to group i, and smaller values of PLD/LCD. Since CN5 is a local-scale descriptor, whereas SASA, PLD, and LCD are medium-scale descriptors, we affirm that the two groups of morphologies are different at both the local and medium scales. In contrast, other descriptors are similar. Thus, these phases are graphitic-like, with mostly six-membered rings, in a way similar to that at 0.50 g/cm^3^, confirming that our selection of the massaging parameters is tailored toward graphitic-like phases. Also, the similar angle distributions A_(sp)_ and A_(sp^2^)_ indicate a similar and regular organization of the carbon atoms within the graphitic planes.

As an illustrative example for comparison with experiment, commercial Ketjen black (KJB, a form of carbon black) is a remarkably interesting material for electroconductive applications. KJB has a bulk mass density of ≈0.10–0.12 g/cm^3^^40^ and average pore size of 7.7 nm,^57^ corresponding to an average pore volume of ≈240 nm^3^, which is close to the largest pore diameter of 6.7 nm in our MM3/MM4 phase, corresponding to a pore volume of ≈161 nm^3^ in Figure S16 (bottom). Note that in other work^88,89^ Ketjen black is modeled, surprisingly, as having a bimodal pore diameter distribution with the smaller-size peak centered around 3 nm and the larger-size peak centered around 30–40 nm, corresponding to a bimodal pore volume distribution with the smaller peak around 14 nm^3^ and the larger peak at more than 14000 nm^3^.

Moreover, a complex intersection of multiwall motifs (graphitic sheets that generate other graphitic sheets by bisections) can be observed in the MM1/MM8 and MM2/MM6 phases in Figure 1 (top row), which may be linked to interesting applications. The appearance of this structure is in good agreement with recent experimental observations of few-wall CNT coils that exhibit single-wall tails;^58^ similarly to that in our model, single graphitic sheets depart from aggregated multiwall regions. This has been suggested to provide interesting properties in terms of electron transport.^58^

The difference between the MM1/MM8, MM2/MM6, and MM3/MM4 phases is also reflected in the corresponding HRTEM images, shown in Figure 4 (top row), which are strikingly different. This suggests that these phases should be easily identified experimentally. We emphasize that the facile generation of such different phases using strictly analogous computational protocols is far from trivial. Moreover, our HRTEM images indicate a crowding of graphitic planes into multiwall features that is reminiscent of a similar crowding present in the experimental HRTEM images from Figure 1c of ref (41). This similarity is more pronounced than with the simulated HRTEM of candidate atomistic structures in the same work. Analogous multiwall signatures can be seen in many experimental studies. For example, HRTEM images of milled carbon soot materials are reported in Figure 1c of ref (90), presenting typical basic structural units of graphitic plane segments: a few nanometers in length and organized into multiwall features reminiscent of the ones observed in our phases. In another work,^91^ soot and carbon black materials were studied *via* HRTEM, finding that the average interplanar distances of the graphene planes ranges around 3.4–3.5 Å, which is larger than the typical plane separation in graphite (3.35 Å), but within the values measured in our phases, *i.e.*, 3.7 ± 0.3 Å. In general, the simulation of the HRTEM images of the generated configurations provides carbon-metric templates with precise dimensions, shapes, motifs, orientations, and reactive regions, which can be compared with experimental data using dedicated software (matching algorithms working on large stores of carbon fingerprints). Moreover, a potentially powerful interpretation of the material characteristics could be obtained through a synergistic combination of DynReaxMas structure generation procedures with algorithms specialized for analysis and processing of HRTEM images, such as the Digital Micrograph (Gatan) program efficiently used by Muller *et al.*(91) to correlate the microstructure of diesel engine soot samples to the predominant bonding and oxygen incorporation.

In conclusion, while our approach needs additional validation, these initial results provide very promising comparisons with experiment. In this connection, we emphasize that similar DynReaxMas massages consistently produce similar carbon morphologies. For example, the presence of a bimodal distribution with one big void and many smaller ones at a mass density of 0.50 g/cm^3^ is present both for the MM3/MM4 massage sequence and for analogous (non-prototyped) massage sequences such as MM3/MM6. Naturally, in amorphous materials there is a massive number of “putative” structures that are homologous but clearly not identical at a very local level. Thus, the detailed way in which similar motifs are distributed in the various regions of the box and differently combined with different extensions does vary from one configuration to the other. This is because these are very complex materials and differ from, *e.g.*, a protein with definite secondary and tertiary structures.

## Conclusions

Here we develop and propose an original DynReaxMas computational methodology (dynamic reactive massaging of the potential energy surface) to explore the phase diagram of amorphous carbonaceous materials. Our approach is based on three pillars:

(i) dynamic reactive PES transformations (also named force-field massaging, FFM);

(ii) global optimization (GO);

(iii) an extensive set of descriptors.

A finely tuned dynamic reactive transformation of the PES or FF-massaging step conducted at a temperature coinciding with the one used in experiment produces a set of structures that we refine using GO stochastic sampling, to be finally identified and classified *via* appropriate descriptors. Importantly, our approach thoroughly explores the complexity of amorphous systems, reflected in the complexity of the force fields called to describe them, by fully exploiting a parallel freedom and complexity of the DynReaxMas procedure in freely tuning the FFM massaging parameters, *i.e.*, the sequence of FF parameters selected for massaging and the extent and length of the corresponding massaging steps.

We applied our methodology to amorphous carbonaceous models in the low-density, graphitic régime using atomistic models of sizes ranging between 4000 and 50000 carbon atoms, with 25000-atom models used for the production runs. We found that this system size is sufficient to generate a variety of morphologies at various densities, therefore enabling interpretation of existing experimental results while potentially triggering the discovery of unexplored phases. Notably, we find that the mass density, traditionally used as the main descriptor of these systems,^2,25,46^ is here qualified as being insufficient to uniquely identify the possible candidate phases. Other descriptors must be included to draw a thorough phase diagram of these systems. Specifically, we found that

(1) the distribution of pore sizes allows one to monitor the competition between more uniform and bimodal distributions of pore volumes, especially effective at mass densities between 1.15 and 0.5 g/cm^3^;

(2) the local density provides a straightforward measure of the competition between agglomerated *vs* sparser phases, with a variable layer thickness of the pore walls;

(3) a further partition can be done into boxed *vs* hollow fibrillar motifs (including CNTs), which is especially effective at a mass density of 0.16 g/cm^3^.

This emerging picture of the evolution of competing phases as a function of the mass density agrees with the general experimental findings of diverse phases at the same density^3,10,32,33,36,41,46,49,79−81,14,16,18,19,23,24,26,28^ and specifically with experimental determinations of local density, pore-size estimates, bimodal-pore-size phases, and intersecting multiwall motifs (including CNTs), as discussed in Results and Discussion.^41,54−58^ Moreover, some phases discovered here, such as the pore-size-bimodal-distribution phase at 0.5 g/cm^3^ with its simultaneous presence of small and large pores and of narrow tunnels, exhibit peculiar features that may lead to properties of interest in various applications.^55,56^

Our final goal is to draw a phase diagram of amorphous carbonaceous materials that catalogs the corresponding typical configurations. That is, we want to provide an atomistic picture of all metastable phases experimentally accessible, in advance of and to be validated by experimental synthesis and characterization. The insight derived and the predictions made can be verified experimentally (work is in progress to further investigate such links with experiment) and might be generalizable to other amorphous materials. Moreover, we emphasize that the facile generation of such different phases using strictly analogous computational protocols is far from trivial but is promising both in terms of efficiency and exhaustive character of the approach.

To achieve this goal, the DynReaxMas methodology enables us to work at a simulated synthesis temperature close to experiment (≈1800–2000 °C). This is crucial, since we show that simulations conducted at high temperatures produce a simplified phase morphology losing much of the pore structure so important for applications (already *T* = 3000 K is above the full graphitization temperature of ≈2800 K,^59^ which holds *a fortiori* for the typical simulations at ≈4000 K, which is on the verge of melting). This is because carrying out simulations at realistic temperatures allows the interplay of activated *vs* entropic processes to be captured correctly (bond breaking and reformation *vs* mass transport and diffusion).

Technically, the computational effort in our approach is reasonable using standard computational facilities and resources.

There are several possible extensions of the methodology developed here for future study. From the materials point of view, we consider here only pure carbon systems. However, passivation of undercoordinated/unsaturated/weak sites and/or edge functionalization with catalysts and sensors represent easy ways to include functional fragments based on oxygen, nitrogen, or transition metal groups while simultaneously stabilizing the system. From the methodological point of view, the automatization of the protocol using recognition techniques (AI) is under study: the DynReaxMas computational methodology or force-field massaging technique is proposed here in a semiautomatic version, but we are working on a fully automatized selection of the sequence of FF massages. Finally, the complete phase diagram achieved *via* the DynReaxMas approach could be further exploited to model dynamic processes such as synthesis, preparation, and growth,^80^ or as an input for reactive global optimization^92^ techniques.

## Methods

### Theoretical Approach and Justification

As mentioned in the Introduction, exhaustive sampling of rugged potential energy landscapes^30,31^ (*i.e.*, energy landscapes exhibiting a myriad of local equilibrium configurations that differ only slightly in energy but are separated by high interconversion barriers due to breaking and re-formation of strong covalent and directional C–C, C=C, and C≡C bonds) represents a challenge to practical computational studies. In this context, both kinetic and stochastic approaches must deal with challenging computational sampling problems.

A rough estimate within the kinetic methodology suggests that an MD simulation lasting 1 ns must be carried out at a temperature of at least 3800 K to overcome the energy barrier for breaking a bond with a strength of 3 eV. Moreover, given the directional features of carbon/carbon binding, randomly positioning a carbon atom adjacent to a given carbon with sp^3^ valence hybridization will have only a 25% chance of landing in the energetically most favorable site, which, multiplied combinatorially by thousands of carbon atoms, makes the number of possible isomers exponentially large with the chance of finding all plausible lowest-energy configurations exponentially negligible. Consequently, typical simulations partition the task of sampling this vast phase space of metastable configurations into two stages: a destructive stage to break carbon–carbon bonds at an extremely high temperature (in the 8000–16000 K range),^39,41^ followed by a reconstructive (annealing) stage in which carbon–carbon bonds develop, still at temperatures^39^ high enough (around 4000 K) to allow fast atomic rearrangement, at the verge of melting. At these remarkably high temperatures of the destructive stage, kinetic approaches will typically be biased toward a subset of high-entropy structures, which for carbonaceous materials are easily recognizable by the dominance of linear chains (combining high translational entropy with strong sp-hybridized bonds). Moreover, the typical simulation temperature for the reconstructive stage (≈4000 K) is well above the full graphitization temperature of ≈2800 K,^59^ making it much higher than experimental (≈2000 K) synthesis of phases with simultaneously graphitic character^43^ and well-defined pore structures so crucial for applications.^44,45^

The implementation of stochastic methods is also problematic for these cases. Within stochastic schemes, the generation of new structures/configurations to be tested *via* random and unbiased searches (such as in standard basin-hopping (BH) algorithms *via* “shake” moves^60^) becomes highly inefficient, making it mandatory to develop more effective strategies.

One possible approach to overcome these issues is to couple kinetic stochastic methodologies^51^ with approaches that artificially (temporarily) deform the PES to reduce energy barriers for isomer interconversion, thereby speeding up exploration of the phase space. Popular techniques along these lines include hyperdynamics^61^ and metadynamics^62^ algorithms. However, these algorithms often provide local, unsystematic explorations that remain confined to the neighborhood of the initial configuration. Therefore, to achieve a thorough sampling, we must (a) transform the PES to reduce dramatically the height of the interconversion energy barriers thereby reducing the simulated synthesis temperature; (b) carry out kinetic simulations sufficiently long to obtain significant transformations away from the initial structure; (c) induce fast convergence onto low-energy isomers within various regions of the phase space; and (d) achieve all this within a manageable computational effort.

We have developed just such an approach to address all these needs, based on the following pillars.

(i) Dynamic Reactive Massaging of the Potential Energy Surface (DynReaxMas): We have devised a variant of MD-driven PES transformations^51,61,62^ dubbed *dynamic reactive massaging* of the PES (DynReaxMas, also named force-field massaging, FFM, for short). The variant implemented here is based on the ReaxFF^32,50^ reactive force field, but the approach is in principle applicable to any reactive force field, including embedded atom models (EAM) or recently developed machine learning force fields.^63^ Our strategy is to focus on those parameters of ReaxFF that, when appropriately *massaged* (tuned), can reduce the interconversion energy barriers below ≈0.8 eV, allowing the system to undertake phase transitions during short (few tens of ps) MD runs at ≈2000 K (*i.e.*, a temperature employed in typical experiments both in the destructive and reconstructive stages^43^ and high enough to accelerate kinetics but still far below the melting temperature of carbon systems (>3800 K) and below the graphitization threshold at ≈2550 K^59^).

(ii) Global Optimization (GO) Searches:^52^ In the present implementation, the GO purely stochastic step is taken at the end of the MD simulation protocol with the goal of refining and locally adjusting configurations generated by the DynReaxMas transformation procedure.

(iii) Multidescriptor Representation: Such complex amorphous materials as the carbonaceous ones may exhibit many potentially metastable phases, which require *independent* structural descriptors to classify and group them into families, a mandatory step to eliminate redundant samples while focusing on representative sets of distinct structures. Here we first consider a large set of descriptors, and then, *for each value of the mass density*, we single out one or two descriptors that distinguish phases having the same mass density produced by the DynReaxMas protocol for a given region of the phase space. The essential idea is that each region of the multidimensional phase space can be characterized by a reduced set of independent descriptors associated with a specific renormalized energy expression on the basis of these descriptors as variables, which is in line with a previous GO approach (Figure 2.9 in ref (53)) and with techniques from machine learning or artificial intelligence.^64−66^

These pillars are realized by a practical step of the methodology, in which the parameters of the approach are selected and tuned.

(iv) PES Transformation and Structure Evolution, Practical Dynamic Reactive Massaging Protocols for Graphitic Carbon: The DynReaxMas procedure is a general approach that has numerous variants. In this exploratory work, we started sampling the vast phase space of protocols with testing and cataloging relationships between force-field massaging and the type of structures generated for the specific case of amorphous carbonaceous systems. Moreover, as a working example, we decided to focus on the low-density régime and graphitic phases, thus specifying and tuning the parameters to optimally investigate the range of graphitizable carbon, which, in terms of applications, corresponds to the range typical of glassy carbon electrodes and carbonaceous deposition supports.

### Dynamic Reactive Massaging of the Potential Energy Surface: Point i

**Figure 5:**
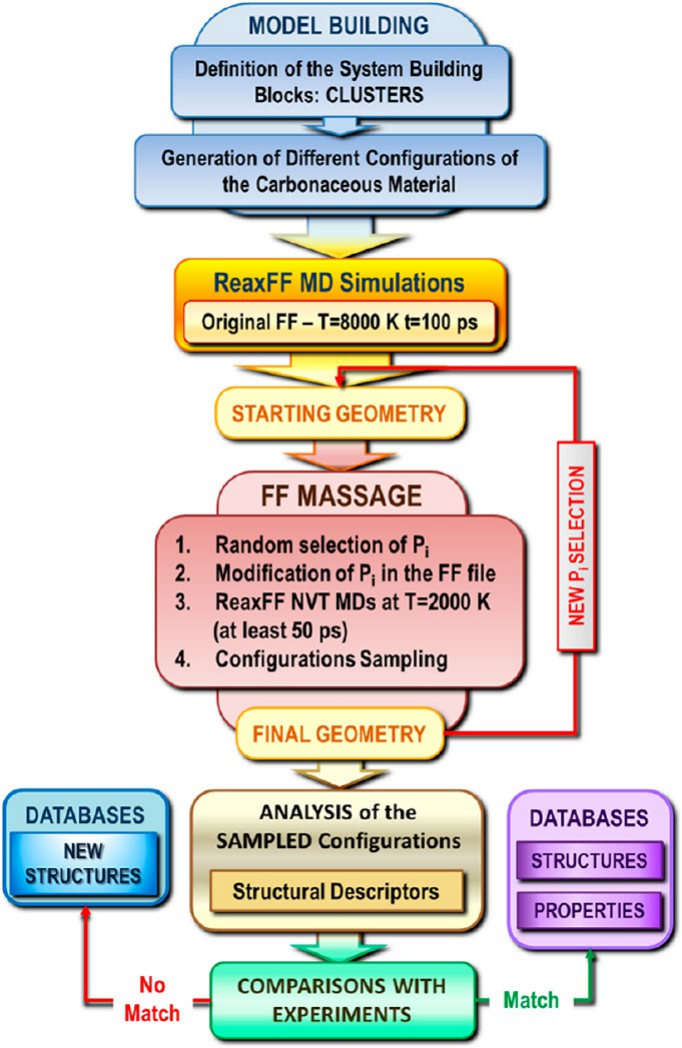
Flowchart of the DynReaxMas approach.

The DynReaxMas approach is illustrated in the flowchart of Figure 5. DynReaxMas simulations were carried out starting from initial configurations created as detailed in the SI. Then, dynamic reactive *massaging* of the PES was used to perturb the PES toward sampling distinct regions of configurational space. All reactive minimizations and molecular dynamics runs were carried out with the ReaxFF code available in the Large-scale Atomic/Molecular Massively Parallel Simulator (LAMMPS) package.^67^ The selected ReaxFF parametrization,^32,50^ C.ff,^68^ has been tuned for carbonaceous systems and validated against experiments and DFT calculations on bulk phases. C.ff accurately describes bulk systems containing carbon coordinated in sp, sp^2^, and sp^3^ hybridization: the formation enthalpy of bulk graphite is predicted to be ≈−175 kcal/mol, in excellent agreement with experiment (−174.8 kcal/mol) and DFT (−178.4 kcal/mol, using the Perdew–Burke–Ernzerhof exchange–correlation functional^69^ augmented with the Grimme-D3 empirical dispersion corrections^70^), and it provides good agreement for the bulk diamond, whose formation enthalpy is predicted to be −174.27 kcal/mol *vs* experiment (−172.93 kcal/mol) and DFT (−175.7 kcal/mol), while the formation enthalpy of an infinite carbon wire (alternating triple and single bonds between sp-hybridized carbon atoms) is reasonably described by C.ff to be −146. kcal/mol *vs* a DFT value of −153. kcal/mol. We emphasize that the DynReaxMas method is general and can be applied to other types of materials or force fields. The present choice of ReaxFF is based on our extensive experience and understanding of the meaning of the FF parameters to obtain sound and effective modulations. We recall, however, that recent rigorous comparative studies^38,39^ show that the ReaxFF parametrization employed here somewhat overestimates the stability of sp-hybridized carbon and that more accurate force fields have been developed recently; see, *e.g.*, ref (63).

The DynReaxMas focused on the force-field parameters reported in Table 2 (see the ReaxFF manual and refs (32, 50, and 71) for a detailed description of the ReaxFF parameters), which were selected after a series of tests to disclose the effects of modifications. A DynReaxMas *massaging step* consists of a series of MD simulations in each of which the ReaxFF parameters were changed sequentially, in the current implementation one at a time. In other words, a *massaging step* consists of an MD run conducted at temperature *T*_1_ for *t*_1_ ps in which the ReaxFF parameter *P*_p1_ is modified by an *M*_m1_*massage* (*e.g.*, −50% reduction), followed by an MD run at *T*_2_ for *t*_2_ ps with the ReaxFF parameter *P*_p2_ changed by an *M*_m2_*massage* (*e.g.*, +30% increase) and so on. A massaging step is then uniquely defined by the number *N* of its *massages* and the sequence of the corresponding massaging parameters {*T*_*i*_, *t*_*i*_, *P*_p*i*_, *M*_m*i*_ with *i* = 1, *N*}. This sequence was engineered in preliminary studies on a 4176-atom model at a density of 1.15 g/cm^3^ (simulation box, 42 × 38 × 45 Å^3^), which allowed us to tune the production procedure and to select force-field parameters that, once modified, most effectively induced phase transformations of the system. Once the FFM sequences were engineered, we then employed them in 25056-atom systems over a range of mass densities between 1.15 and 0.16 g/cm^3^, as described below (test simulations were also conducted on 50112-atom systems and are available upon request). For the FFM *M*_*i*_*massages,* we used a reduction of the ReaxFF parameters to half of their original values because this usually proved to be the best option. This recipe will be generalized in the future to randomly select the {*M*_*i*_} and to find the most appropriate values for each {*P*_*i*_}.

**Table 2:** Force-Field Parameters Selected for the PES Transformation (*Massaging*) Simulations

force-field section	param identifier	param FF name	description	field position
ATOM	P1	Evdw [1]	van der Waals dissociation energy	5
	P2	Eunder [2]	undercoordination energy	12
BOND	P3	Edis1 [3]	*D*_e_^σ^ sigma-bond dissociation energy	1
	P4	Edis2 [4]	*D*_e_^π^ pi-bond dissociation energy	2
	P5	Edis3 [5]	*D*_e_^ππ^ double pi-bond dissociation energy	3
	P6	pbe1 [6]	bond energy	4
	P7	kov[7]	overcoordination penalty	8
	P8	pbe2 [8]	bond energy	9
ANGLE	P9	theta0 [9]	equilibrium angle	1
	P10	ka [10]	1st force constant	2
	P11	kb [11]	2nd force constant	3
	P12	pv3 [12]	energy/bond order	7
TORSION	P13	V1 [13]	V1-torsion barrier	1
	P14	V2 [14]	V2-torsion barrier	2
	P15	V3 [15]	V3-torsion barrier	3

### Global Optimization Searches: Point ii

Global optimization (GO) runs are conducted at the end of the simulation protocol. The goal of these simulations is not phase generation but to search for the local minima in a neighborhood of the structures produced *via* the DynReaxMas massaging protocol. GO runs are performed *via* a basin-hopping (BH) algorithm^60^ according to the following procedure: First, local exploration of the phase space is obtained *via* a PES transformation linking each point to its nearest local minimum through a steepest-descendent algorithm. Second, a neighborhood of the local minimum is explored *via* a short (0.25 ps) MD at *T* = 2000 K on a transformed PES based on a *gently massaged* force field obtained by rescaling the *P*_9_ parameter (see Table 2) to 90% of its original value. Then, third, another short (1 ps) MD (from 2000 to 300 K) based on the nonmassaged force field is carried out. Finally, fourth, the last sampled configuration is minimized and accepted according to a Metropolis criterion^60^ with an energy acceptance threshold of 0.5 kcal/mol per carbon atom.

### Multidescriptor Representation: Point iii

A proper set of independent structural descriptors is necessary to recognize potentially unfamiliar phases of amorphous materials. Our strategy is to consider initially a comprehensive set of phase-space descriptors and then, *for each value of the mass density*, to single out within this wide set of descriptors those few that are effective in that given region of the PES, in order (iii-a) to characterize the different phases found and (iii-b) in perspective, to boost the GO search when using the descriptors to “repel” walkers within the parallel excitable walker basin-hopping (PEW-BH) approach.^60^

In addition to mass density (the main descriptor), we considered the following descriptors:

(1) *C*_1_: This is the fraction of C atoms with coordination 1. Coordination is defined as an integer number and corresponds to the number of C neighbors within a sphere of radius 1.8 Å.

(2) *C*_2_: This is the fraction of C atoms with coordination 2. *C*_2_ atoms are sp-hybridized (typically, in wires alternating single and triple bonds) or sp^2^-hybridized but undercoordinated.

(3) *C*_3_: This is the fraction of C atoms with coordination 3. *C*_3_ atoms are sp^2^-hybridized (typically, organized in graphitic sheets) or sp^3^-hybridized but undercoordinated.

(4) *C*_4_: This is the fraction of C atoms with coordination 4. C atoms in this category are sp^3^-hybridized (and involved in the formation of diamond-like 3D tetrahedral architectures).

(5) *A*_(sp)_. This is the average difference in degrees between the ideal 180° angle for sp-hybridized C atoms and actual angles formed by *C*_2_ atoms. Large values correspond to “bent wires” deviating from ideal linearity and therefore partly sp^2^-hybridized.

(6) *A*_(sp^2^)_. This is the average difference in degrees between the ideal 120° angle for sp^2^-hybridized C atoms and actual angles formed by *C*_3_ atoms. Large values correspond to “warped sheets” deviating from perfect planarity of ideal graphitic foils and therefore partly sp^3^-hybridized.

(7) *R*_5_: This is the ratio between the number of five-membered rings in the structure and the total number of atoms in the cell. Rings are identified *via* the “rings” code.^29^

(8) *R*_6_. This is the ratio between the number of six-membered rings in the structure and the total number of atoms in the cell.

(9) *R*_7_. This is the ratio between the number of seven-membered rings in the structure and the total number of atoms in the cell.

(10) CN_5_. This is the integral of the pair distribution function *g*(*r*) from 0 to 5.0 Å (up to second neighbors) calculated by the *rings* code: this quantity is a measure of the local density of the system.

(11) SASA. Solvent-accessible surface area of the structure (water probe, −1.4 Å) is calculated by the GROMACS code in Å^2^.^72,73^

(12) *D*_av_. Average density of the system, in g/cm^3^, is calculated by GROMACS from the SASA of the structure.

(13) FV. Fraction of free volume in the system is calculated by GROMACS (water probe).

(14) PSD. pore-size distribution is calculated by the PoreBlazer4.0 code as a function of the pore radius in Å.^74^

(15) PLD. Pore limiting diameter, in Å, corresponds to the largest probe that can cross the simulation cell in at least one dimension *via* a diffusive pathway, calculated by the PoreBlazer4.0 program.^74^

(16) LCD. Largest cavity diameter, in Å, corresponds to the largest pore in the structure, calculated by the PoreBlazer4.0 code.^74^

(17) *S*_AC,T_. Total accessible surface area, in m^2^/g, corresponds to the total accessible surface for nitrogen gas, mimicking a real adsorption experiment, calculated by the PoreBlazer4.0 code.^74^

(18) *S*_AC,A_. Network accessible surface area, in m^2^/g, corresponds to the accessible surface area obtained specifically for a nitrogen-accessible network, calculated by the PoreBlazer4.0 code.^74^

(19) *V*_PO,T_. Total probe-occupiable volume, in cm^3^/g, corresponds to the volume defined by the surface calculated according to the total accessible surface (*S*_AC,T_), calculated by the PoreBlazer4.0 code.^74^

(20) *V*_PO,A_. Network accessible probe-occupiable volume, in cm^3^/g, corresponds to the volume defined by the surface calculated according to the network-accessible surface (*S*_AC,A_), calculated by the PoreBlazer4.0 code.^74^

(21) *F*_He,T_. Helium probe volume fraction is calculated by the PoreBlazer4.0 code.^74^

(22) *E*_av_. Average energy per carbon atom is used in the Metropolis criterion of the BH algorithm and is an important parameter to monitor structural stability.

We also employed the CAVER code for further graphics^75^ (see below). Naturally, other descriptors are possible as employed in recent work.^40^ We also use the root-mean-square deviation (RMSD) and the root-mean-square fluctuation (RMSF), measuring the deviation of a configuration from a reference structure and the time average of the deviation from an average value, respectively. The definition of these standard quantities is summarized for convenience in the Supporting Information. Finally, high-resolution transmission electron microscopy (HRTEM) images corresponding to the generated configurations were simulated using the QSTEM software,^76,77^ which is based on the multislice method originally proposed by Cowley and Moodie.^78^ The parameters used in the simulations were as follows: number of slices = 20, slice thickness = 5 Å, 200 kV extraction voltage, 1.0 mm spherical aberration coefficient, and −60.0 nm defocus value (Scherzer defocus). The 20 selected depths are indicated by the focal plane.

### PES Transformation and Structure Evolution, Practical Massaging Protocols for Graphitic Carbon: Point iv

To turn the general DynReaxMas approach into a practical tool, the link between the FFM parameters and the morphology of generated structures must be understood and then specified to the system of interest. Here we start sampling the vast phase space of DynReaxMas protocols, deepen our understanding of the possibilities of the approach, arrive at a few operative DynReaxMas procedures, and devise three protocols aimed explicitly at studying graphitic carbonaceous materials, thus enabling the applications discussed in Results and Discussion.

**Figure 6:**
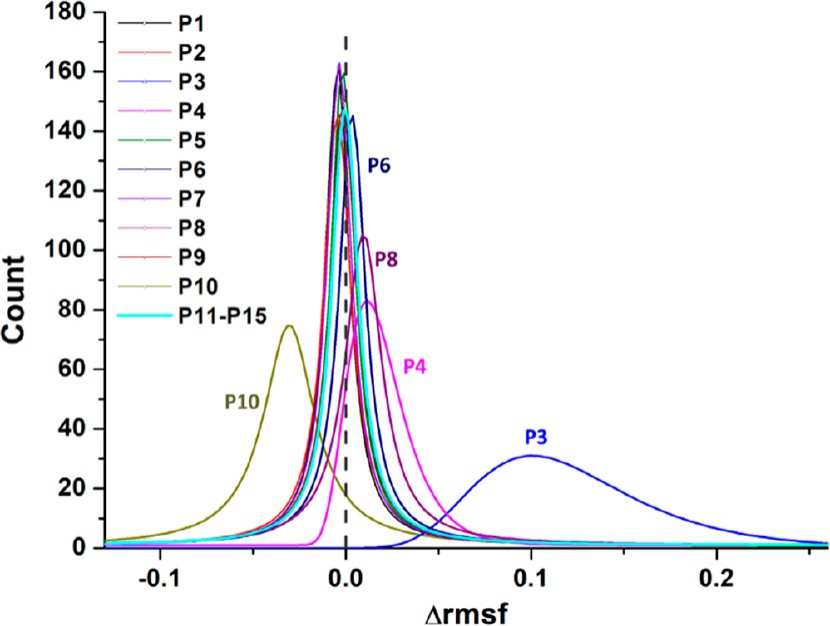
Atomic fluctuations (nm) after massaging of the ReaxFF parameters (50% reduction relative to the original ReaxFF parameter).

First, to estimate the effects of the modulation of each parameter, we carried out 15 single-parameter single-step DynReaxMas *massaging* MD simulations at 2000 K on a 4176-atom model at a density of 1.15 g/cm^3^, starting from the same initial geometry, for approximately 20 ps, and analyzed the atomic fluctuations induced by the force-field modifications, *i.e.*, the RMSF of the carbon atoms (RMSF distributions are reported in Figure 6). As apparent from an inspection of Figure 6, all *massages* had an impact on both atomic fluctuations and structure (Table S1 and Figures S1 and S2), and produced a great variety of atom assemblages, suggesting that the selected parameters, containing both *hard* and *soft* degrees of freedom, are appropriate for exploring the PES of carbonaceous systems. The *hard* degrees of freedom are those whose massaging produces the largest fluctuations associated with parameters such as P_3_, P_4_, and P_10_, *i.e.*, energies and force constants of the chemical bonds. The *massage* effects were more marked when massaging the *hard* degrees of freedom, producing, in the most impressive example, a disrupted configuration mainly made of short carbon chains (filaments); see Figure S2, P_3_. In contrast, the modulation of *soft* degrees of freedom biased the evolution of the systems to a lesser degree, favoring, in some instances, a fast formation of nanotube-like structures (Figure S2, P_4_). These findings suggest that an appropriate combination of *hard* and *soft massages* can lower the barriers to bond breaking, making the system transition between diverse regions of the PES (*hard massages*), and then reduce the bond re-formation barriers (*soft massages*) to obtain a fast convergence to stable local arrangements. In other words, as in traditional simulations,^38,39,41^ we divide the task of structure generation into two successive stages: a first destructive stage to break carbon–carbon bonds, in which we use *hard massages*, and a second reconstructive stage to form carbon–carbon bonds, in which we use *soft massages*. Note that, at variance with traditional simulations, we consistently use a uniform simulation temperature close to that used in experiment for both destructive and reconstructive stages: we do not need to work at unphysically high temperatures because our kinetics are accelerated by the massaging tool.

To test this strategy, we defined and applied the random biasing generation schemes shown in the flowchart of Figure 5, based on the repetition of a given {*T*_*i*_, *t*_*i*_, *p*_*i*_, *M*_*i*_, with *i* = 1, *N*} manipulation sequence composed of both *hard* and *soft massages*. To begin with, we set *N* = 4 and explored ten combinations (listed in Table S2) of randomly generated {*N* = 4} *massages*, denoted as MM*n* with *n* = 1–10, all consisting of 200 ps *NVT* MDs at 2000 K (the length of the *i-massaging* of each parameter was fixed to 50 ps). The induced perturbation to the structure was monitored by examining the RMSD of the carbon atoms with respect to the starting configuration (Figure S3) and comparing with the RMSD measured during an MD simulation based on the *nonmassaged* force field, which is around 9 Å on average. The structural rearrangement is already significant after the first step (RMSD > 9.0 Å), and the trend/amount of the deviations is in line with the nature of the modified parameter. In most cases, the RMSD does not change after the first perturbation, but this is not the case for MM1, MM3, and MM7, where the final values of RMSD are almost doubled (MM2 could also be included in this set, to a more limited extent). These *massages,* in fact, contain perturbations of *hard* parameters, namely, *P*_9_ and *P*_3_, *i.e.*, the equilibrium angle and the energy of the σ bonds, respectively, that considerably destabilize the characteristic conformations of the carbon atoms.

The RMSD can give a sense of the structural transformations, but alone it is not sufficient to visualize the final geometry into which the system has evolved. To classify the sampled configurations, we used the set of descriptors defined at point iii to discriminate among the various structures. The values of the descriptors for the final geometries before and after the GO step are reported in Table S3. The data of Table S3 show that the role of GO after the DynReaxMas *massaging* protocol consisted of slight local relocations of the atoms, as indicated by small changes of the angular descriptors, but did not modify the whole structures appreciably, as intended.

The effect of the sequential massages MM*n* instead produced an assortment of configurations that could be grouped into four sets.

(1) The most numerous set contains six members, namely, MM4, MM5, MM6, MM8, MM9, and MM10, and is characterized by a high percentage of *C*_3_ (94% at most), a low percentage of *C*_2_ (14% at most), and a high number of five- and six-membered rings. The average local density is around 9–12 g/cm^3^, implying that the atoms are not too tightly packed, and empty regions are distributed in the cell, separating the structured regions of the material. All these features agree with an extended graphitized-sheet geometry with bent and rolled conformations (a typical one is shown in Figure S4, MM4).

(2) A second set contains only two members, namely, MM1 and MM3, and is characterized by a substantial presence of *C*_3_ (65% on average), a tangible presence of *C*_2_ (34% on average), fewer six-membered rings than in the more populated family, and a lower average density. This suggests the simultaneous existence of sheet regions and connecting filaments (Figure S4, MM3; see also Figure S6, MM1, MM3).

(3) The other two configurations are mostly made of filaments differently packed and organized: in MM7, the packing is very tight; 96% of the carbons have sp-hybridization and are arranged in wires alternating single and triple bonds, whereas in MM2 coiled chains are randomly distributed inside the box (Figure S6, MM2 and MM7).

The results of this training exercise confirm our strategy/hypothesis that an efficient exploration of the phases space should contain *hard* parameter perturbations at the beginning of the process (in the destructive stage) but not at the end (in the reconstructive stage), and a balanced combination of the other perturbations mixing those massages that have produced structures belonging to the two most numerous sets. In connection with the next paragraph, we note that MM1, MM2, and MM3 are appealing candidates for the destructive stage since they generate graphitic phases while producing either a significant presence of *C*_2_ atoms (MM1, MM3) or else randomly distributed coiled chains (MM2) that are more likely prodrome of extended sheets than tightly organized filaments (MM7).

To turn this information into an operative tool tuned for a specific working case, we decided to focus on the low-density régime of carbonaceous materials. This choice is justified by the technological importance of graphitizable carbon in catalytic, sensing, and electrochemical applications, as well as the expectation that carbonaceous materials exhibit a rich and only partially explored phase diagram in this low-density régime. This is justified by the wide variety of experimental results and the number of experimental phases discovered but still only partially unveiled.^3,10,32,33,36,41,46,49,79−81,14,16,18,19,23,24,26,28^

We, therefore, selected three sequences (MM1, MM2, and MM3) containing *hard* ReaxFF parameters (for the rationale of this choice, see the previous paragraph) and three sequences (MM4, MM6, and MM8) containing *soft* ReaxFF parameters. We then switched from the smaller 4176-atom system to a production-phase larger 25056-atom system and performed DynReaxMas runs with all nine possible combinations MM*i*/MM*j* (with *i* = 1, 2, 3; *j* = 4, 6, 8) = {MM1, MM2, MM3} × {MM4, MM6, MM8} for each value of mass density of interest (*i.e.*, 0.16, 0.50, and 1.15 g/cm^3^; see Results and Discussion), for a total of 27 DynReaxMas runs (to enhance the statistical sampling of the different levels of graphitization). We report in the SI (Tables S4–S6 and Figures S8–S10, S14–S16, and S18–S20) a complete set of results from these 27 simulations. Moreover, we found from these simulations that three combinations (MM1/MM8, MM2/MM6, and MM3/MM4) produce diverse and representative phases. Thus, we propose prototyped DynReaxMas massages to optimally investigate this class of materials. The corresponding results are reported as production runs in the main text. The prototyped DynReaxMas perturbation sequences or massages are the following:
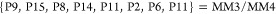
1
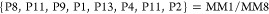
2
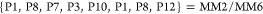
3where *T* = 2000 K, all the {*M*_*i*_} are −50% (as anticipated), and the MD times are *t*_*i*_ = 50 ps. In Results and Discussion, we present the results of these three typical massaging sequences. In the production runs we study systems consisting of a larger number of atoms than the 4176-atom model considered in the exploratory runs. It should be stressed that, as a rule, the difficulty in achieving equilibration increases with the size of the system (*e.g.*, *t*_*i*_ = 120 ps is needed for 50112-atom systems).

A rationale for the choice of the three prototyped massages and some insight into how the DynReaxMas protocol manages to achieve accelerated dynamics can be derived from plots of descriptors as a function of time during the DynReaxMas steps reported in Figure S11 at a mass density of 0.5 g/cm^3^. These plots are also meant to provide indications to DynReaxMas users on how to tune the massaging to explore the desired region of the phase space. Figure S12 shows contour plots obtained from the time evolution of selected parameters during the prototyped DynReaxMas massages: MM1/MM8, MM2/MM6, and MM3/MM4 (at a mass density of 0.5 g/cm^3^) and highlights the correlation among the number of *C*_3_ atoms, six-membered ring content (*R*_6_), and local density, which further supports our choice of prototyped FFM massages. This picture shows how the selected DynReaxMas massages sample mostly graphitic phases as intended. Indeed, it can be noted how the percent of *C*_3_ carbons is always dominant, but *with a different width* and in different regions of other descriptors (in the present case, the number of six-membered rings and the local density).

Note that, to ensure the stability of the produced structures, we added an equilibration step to our phase generation protocol. Thus, after the DynReaxMas MM*i*/MM*j* destructive/reconstructive massages at the chosen temperature (here, 2000 K), we equilibrated the resulting configurations at 2000 K using the original (unbiased, not-massaged) ReaxFF (C.ff) for 50 ps, before finally performing a global optimization run as a final refinement step. All the production results thus refer to phases obtained *via* DynReaxMas MM*i*/MM*j*, followed by equilibration, followed by GO.

Finally, to show the convergence of our protocol, we report in Figure S5 atomic RMSD over the last 10 ps of the equilibration step (left panel) and one example of the superposition of the average and snapshot atomistic structure in the same equilibration step (right panel) for all nine DynReaxMas MM*i*/MM*j* massages at a mass density of 0.50 g/cm^3^. These results show that no qualitative transitions occur during the equilibration step.

To recapitulate, our strategy to define DynReaxMas protocols consists of the following steps:

(i) sample the effect of massaging single ReaxFF parameter in preliminary tests (here, on 4176-atom systems);

(ii) single out ReaxFF parameters to be massaged in *hard* (destructive) and *soft* (reconstructive) stages;

(iii) generate (here, random) MM*i* sequences of a given number (here, 4) parameter massages containing at most one *hard* parameter and analyze the configurations produced by them;

(iv) in view of the region of the phase diagram to be investigated (here, graphitic phases), select a number (here, 3) of appropriate MM*i* sequences for the destructive stage and a number (here, 3) of appropriate MM*i* sequences for the reconstructive stage, respectively;

(v) perform all combinations of destructive and reconstructive MM*i*;

(vi) analyze the results.

Naturally, several other strategies are possible and will be explored in future work.

### Role of the Simulation Temperature

The results presented so far all refer to a simulation temperature of 2000 K. We justify this choice as follows. Focusing on a mass density of 0.50 g/cm^3^, most common in the applications related to carbonaceous materials, we conducted DynReaxMas simulations using four different massages (MM1/MM4, MM1/MM8, MM3/MM4, MM3/MM8) and four different temperatures (1500, 2000, 2500, and 3000 K), and compared the resulting structures. We then report in the SI the following:

(1) in Figure S7 atomistic depictions of all final configurations obtained after different DynReaxMas massages;
(2) in Figure S13 the PSDs of the same final structures;
(3) in Figure S17 simulated HRTEM images of the subset of final structures obtained at 3000 K;
(4) in Figure S21 plots of pair distribution functions (PDFs) or *g*(*r*) of all the final configurations;
(5) in Videos S1, S2, S3, S4, S5, and S6 atomistic movies as a function of time of the final reconstructive stage (*i.e.*, MM8 or MM4, run after MM1 or MM3, respectively).

The above information illustrates clearly how the morphology of the resulting amorphous carbon material depends strongly on the simulated synthesis temperature. We remark that simulations conducted at the highest temperature of 3000 K invariably present more graphitized but also sparser phases, in which most of the complex pore structure obtained at lower temperatures is lost or at least appears very simplified. This is best appreciated in the atomistic pictures of Figure S7, in the simulated HRTEM images of Figure S17, and in the atomistic movies depicting the evolution of the system during reconstruction, with the PSD and PDF of Figures S17 and S21 that perfectly support this picture: thus, in the plots of Figure S21 the first peak in the PDF gives a measure of the short-range graphitic character, which is dominant by construction in our phases; but interestingly, the height of this peak decreases when working at 3000 K instead of 2000 K. This result is in excellent agreement with and rationalizes the experimental finding that ≈2000 K (around 1800–2000 °C) is needed to obtain phases with a dominant graphitic character,^43^ but one should work below the full graphitization temperature of 2550 K^59^ to avoid losing a well-defined pore structure which is crucial for applications, as discussed in refs.^44,45^ We thus demonstrate the crucial importance of using an accelerated dynamics algorithm at a temperature coinciding with the one employed in experiment,^43^*i.e.*, a temperature high enough to accelerate kinetics but still far below the melting temperature of carbon systems (>3800 K). These findings demonstrate the importance of simulation approaches such as DynReaxMas that transform the PES to reduce dramatically the height of the interconversion energy barriers and therefore the simulated synthesis temperature, while also providing microscopic insight and strong theoretical support to the current experimental thrust in the search of synthesis protocols working at lower temperatures to attain a diversity of graphitic phases.

### Perspective Developments

In addition to the results presented here, we highlight the observation that the present work offers interesting perspectives, and several algorithmic and system extensions and developments can be envisioned.

From the algorithmic point of view, alternative but related approaches can be easily imagined. For example, a pure GO approach using other forms of shake moves (alternative to the single-parameter “massaging” technique) represents an exciting alternative to the protocol explored here. The GO stochastic sampling needs efficient moves that perturb the system significantly to carry it to different regions of the PES. These steps should not be so destructive that memory of the PES region in which the system is currently located is lost. Another possible modification of the DynReaxMas algorithm is through *massaging* more than one FF parameter simultaneously. This differs from the sequential parameter massaging employed in this work and could exploit the many-body character and inter-relationships among different FF parameters to produce even more diverse moves. Figure 6 is a good starting point for these alternative approaches since it provides a qualitative picture of the parameter space, *i.e.*, of the correspondence between a given parameter massaging and a given descriptor.

From the system point of view, we considered here exclusively pure carbon materials. However, the present results enable further developments, such as the introduction of additional elements (oxygen, nitrogen, and transition metals, *etc.*) into the carbonaceous framework. This can be done through (i) passivation of low-coordinated sites and/or (ii) edge functionalization:

(i) Despite the thoroughness of our GO search, residual low-coordinated sites with unsaturated dangling bonds are present in our final configurations, produced inherently by stochastic methods,^52^ as quantified briefly in Results and Discussion. These sites are chemically reactive and could act as weak spots for material degradation.^79,82,83^ Passivation of such a reactive site with either hydrogen or oxygenated groups (OH, COOH, and so on) is an effective way to remove weak spots, simultaneously mimicking the real synthesis and stabilization process of the material;

(ii) The graphitic leaflets which abound in our materials and their edge termination can be functionalized with more complex oxygenated, nitrogenated, and so on residues that can act as anchoring points where transition metal atoms or more complex species can attach and impart to the carbonaceous materials catalytic or sensing properties.^12−15^

## Supporting Information Available

Additional computational details; force-field parameters for PES transformation (massaging) simulations; root-mean-square deviations between sampled geometries, their schematic depiction, and values of structural descriptors; convergence check; atomistic depictions, pore-size distributions, and simulated HRTEM images; evolution of selected descriptors; plots of pair distribution functions; depiction of a narrow tunnel (PDF)
Video S1 showing atomistic movie of MM1/MM8 final reconstructive stage at 1500 K as function of time (MP4)
Video S2 showing atomistic movie of MM1/MM8 final reconstructive stage at 3000 K as function of time (MP4)
Video S3 showing atomistic movie of MM1/MM8 final reconstructive stage at 2000 K as function of time (MP4)
Video S4 showing atomistic movie of MM3/MM4 final reconstructive stage at 1500 K as function of time (MP4)
Video S5 showing atomistic movie of MM3/MM4 final reconstructive stage at 2000 K as function of time (MP4)
Video S6 showing atomistic movie of MM3/MM4 final reconstructive stage at 3000 K as function of time (MP4)
