# Supplementary material for: Diverse Phases of Carbonaceous Materials from Stochastic Simulations
Source: ACS Nano. 2021 Mar 15;15(4):6369–85. doi: 10.1021/acsnano.0c08029 (PMC9639862; doi:10.1021/acsnano.0c08029)
Supplement: Supplementary file 1 — nn0c08029_si_001.pdf [file nn0c08029_si_001.pdf]

Supporting Information for
"Diverse Phases of Carbonaceous Materials from Stochastic Simulations"

Susanna Monti[1,†], Giovanni Barcaro[1,†], William A. Goddard[2], Alessandro Fortunelli[1,2*]

[1]*CNR-ICCOM & IPCF, Consiglio Nazionale delle Ricerche, ThC2-Lab and Molecular Modelling Team, via Giuseppe Moruzzi 1, 56124 Pisa, Italy*

[2]*Materials and Process Simulation Center (MSC), California Institute of Technology, Pasadena, California 91125, United States*

[†] Equally contributing first authors

[*] Corresponding author – e-mail: alessandro.fortunelli@cnr.it

Computational Details

All reactive molecular dynamics runs were carried out with the ReaxFF code available in the Large-scale Atomic/Molecular Massively Parallel Simulator (LAMMPs) package.[1] Periodic boundary conditions were applied in all directions, and the time step was set to 0.2 fs. The temperature was controlled through the Berendsen's thermostat[2] with a relaxation constant of 0.1 ps.

Generation of initial configurations

To start the ***PES Transformation and Structure Evolution – point (i)*** initial carbon configurations were created by randomly dispersing small carbon clusters inside a cubic simulation box where the size was determined based on the chosen density. The clusters were further randomized, to create unbiased arrangements, by disassembling their atoms at high-temperature (8000 K) in the NVT ensemble, in a period of about 100 ps. Then, the final configurations were used as starting structures of a series of short (around 100 ps each) MD simulations based on a variety of force fields, with modified parameters, at a temperature (2000 K) lower than the graphitization threshold (approximately 2550K). The total simulation time (1 ns, at most) was sufficient to obtain relatively stable arrangements.

Estimate of cpu time

We use a Linux cluster: 1 node with 96 cores (see below), cpu GHz = 2.3.
We run 100 ps of MD, time step 0.2 fs, saving structures every 1000 steps (0.2 ps), total number of collected snapshots = 500, whence a total cpu time ≈ 13 h.

Architecture:	x86_64
CPU op-mode(s):	32-bit, 64-bit
Byte Order:	Little Endian
CPU(s):	96
On-line CPU(s) list:	0-95
Thread(s) per core:	2
Core(s) per socket:	12
Socket(s):	4
NUMA node(s):	4
Vendor ID:	GenuineIntel
CPU family:	6
Model:	85
Model name:	Intel(R) Xeon(R) Gold 5118 CPU @ 2.30GHz
Stepping:	4
CPU GHz:	2.3

RMSD and RMSF Quantities

The root mean square deviation (RMSD) measures the deviation of a structure from a reference structure (RMSD=0.0 indicates a perfect overlap). The RMSD is defined as:

$$RMSD = \sqrt{\frac{\sum_{i=0}^{N}[m_i * (X_i - Y_i)^2]}{M}}$$

where N is the number of atoms, m_i is the mass of atom *i*, X_i is the coordinate vector for target atom *i*, Y_i is the coordinate vector for reference atom *i*, and M is the total mass. If the RMSD is not mass-weighted, all m_i= 1 and M = N.

The root mean square fluctuation (RMSF) of a structure is the time average of the deviation of a structure from its average value. It is calculated according to the equation below, where x_i is the coordinates of particle i, and $\langle x_i \rangle$ is the ensemble average position of i:

$$RMSF= \sqrt{\langle (x_i - \langle x_i \rangle)^2 \rangle}$$

The RMSD quantifies how much a structure diverges from a reference over time, the RSMF reveals which areas (atoms) of the system are the most mobile. While RMSD is frequently calculated with respect to the initial state, the RMSF is calculated with respect to an average structure of the simulation. An area of the structure with high RMSF values frequently diverges from the average, indicating high mobility.

Note that we compute RMSD and RMSF after a rigid-body optimal alignment of the atomic coordinates in each snapshot to a reference structure (starting or average configuration).

Definition of massaging protocol

The parameters we have chosen to explore in the 'massaging' procedure, reported in Table 1, belong to the atom, bond, valence angle and torsion sections of the ReaxFF force field, which is divided into 7 parts, containing the general, atom, bond, off-diagonal, valence angle, torsion angle, and hydrogen bond parameters – we refer to the ReaxFF manual[3,4,5] for a detailed description. As explained in the ReaxFF manual, all parameters without physical meaning are named after the partial energy contribution (valp1, vapl2, *etc.*). In contrast, the other ones, like the torsional rotational barriers (V1, V2, V3), have names connected to physical quantities or descriptors.

Table S1. Force field parameters selected for the PES transformation (*massaging*) simulations (50% parameter reduction). Root mean square deviations between the initial and final sampled geometry are reported in the last column. Parameters' names and descriptions have been extracted from the ReaxFF manual.

Force Field Section	Parameter Identifier	Parameter FF Name	Description	RMSD (Å)
**ATOM**	P1	eps	vdW dissociation	8.7
	P2	valp1	En. Undercoord. En.	8.1
**BOND**	P3	de1	D_e^{σ}	15.9
	P4	de2	D_e^{π}	11.3
	P5	de3	$D_e^{\pi\pi}$	9.1
	P6	psi	pbe1, bond En.	8.5
	P7	vover	overcoord. penalty	8.4
	P8	psp	pbe2, bond En.	9.0
**ANGLE**	P9	th0	eq. angle	8.6
	P10	vka	force const.	7.6
	P11	vka3	force const.	9.8
	P12	vval	En./bond ord.	9.3
**TORSION**	P13	V1	barrier 1	9.0
	P14	V2	barrier 2	8.9
	P15	V3	barrier 3	9.3

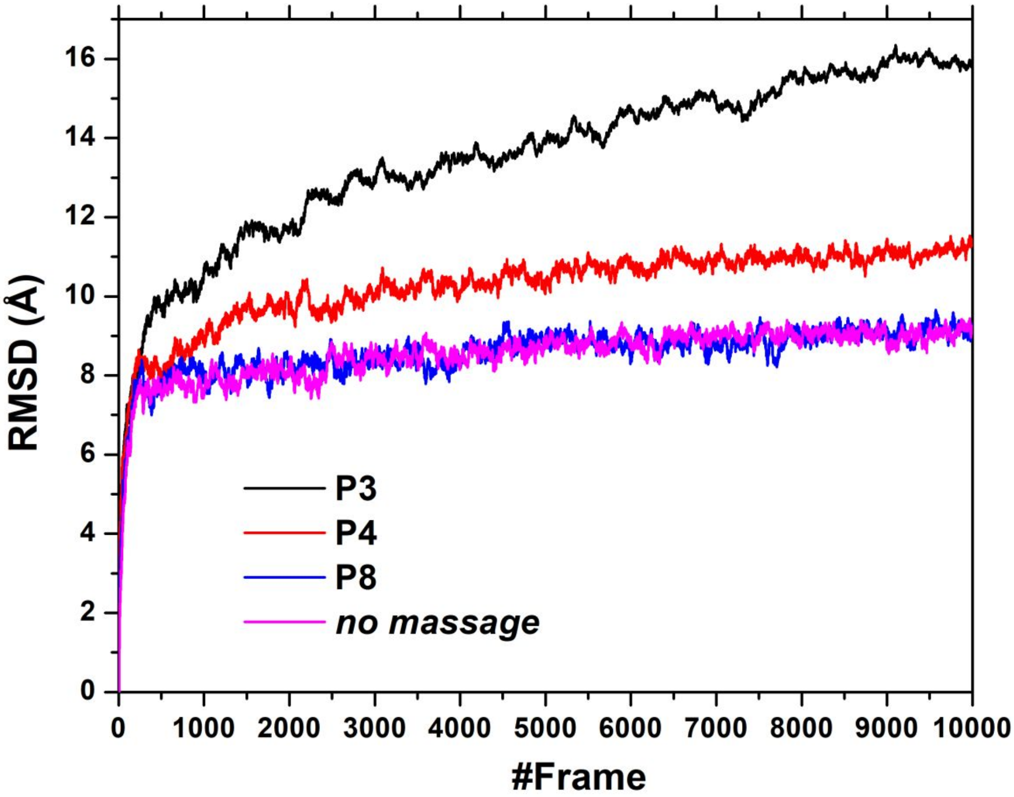

Figure S1. Root mean square deviation of all the atoms of the sampled configurations relative to the initial structure. The chosen prototype system contains 4176 atoms (simulation box = 42 × 38 × 45 Å^3). NVT MD at 2000 K for 20 ps, structures are collected every 2 fs.

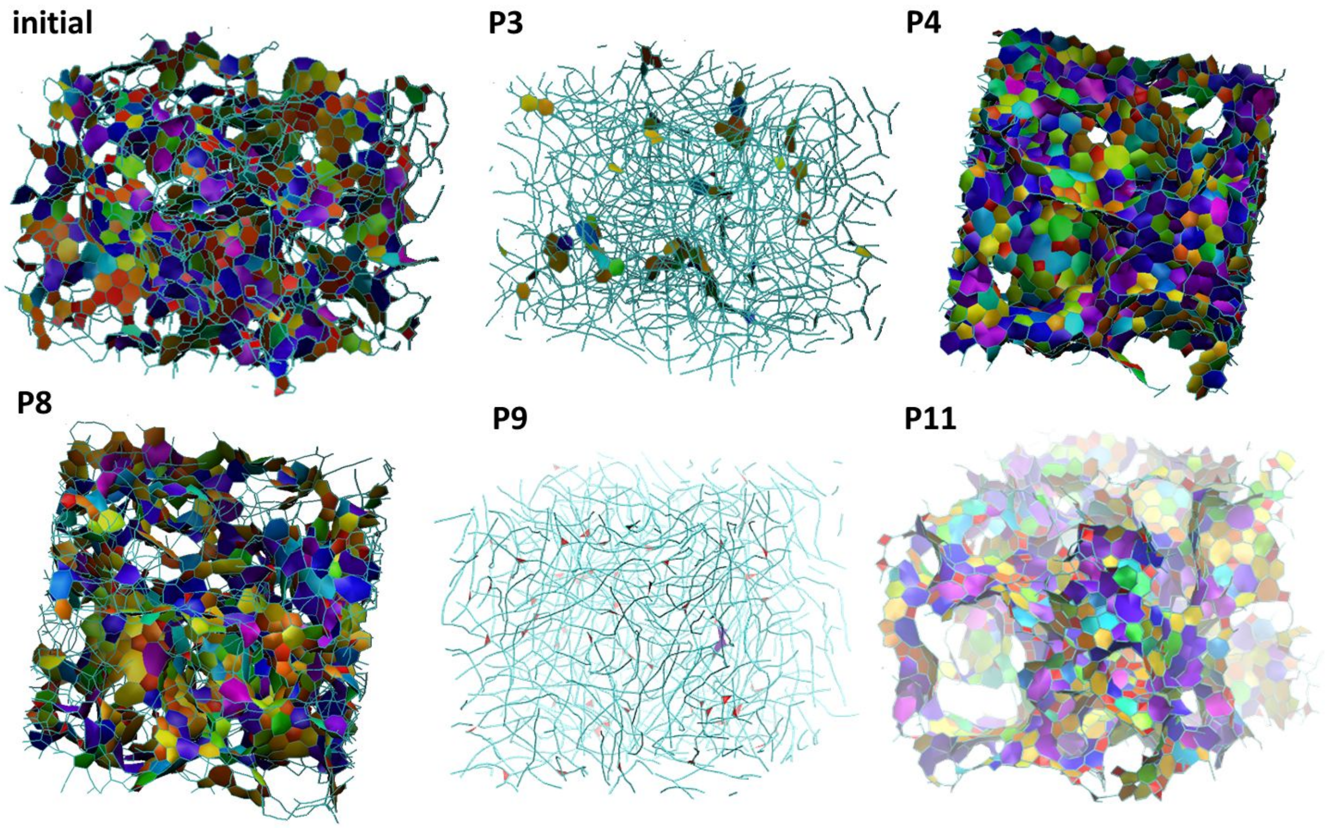

Figure S2. Initial and final structures obtained after 20 ps *massaging* MD at T=2000 K in the NVT ensemble. The *massaged* parameters are indicated with P_i in each case, and the molecular structures are represented with cyan lines connecting the carbon atoms and filled colored rings. Different colors are used to distinguish the number of ring members. The initial structure is the same prototype system, containing 4176 atoms (simulation box = 42 × 38 × 45 Å^3), cited in Figure S1.

Table S2. Force field parameters randomly selected for the four-step-*massage* NVT MD simulations at T=2000 K (50% parameter reduction), starting from the small prototype model of a carbonaceous material.

MM1	MM2	MM3	MM4	MM5	MM6	MM7	MM8	MM9	MM10
P8	P1	P9	P11	P12	P10	P7	P13	P4	P15
P11	P8	P15	P2	P6	P1	P15	P4	P4	P11
P9	P7	P8	P6	P14	P8	P13	P11	P7	P4
P1	P3	P14	P11	P5	P12	P9	P2	P7	P2

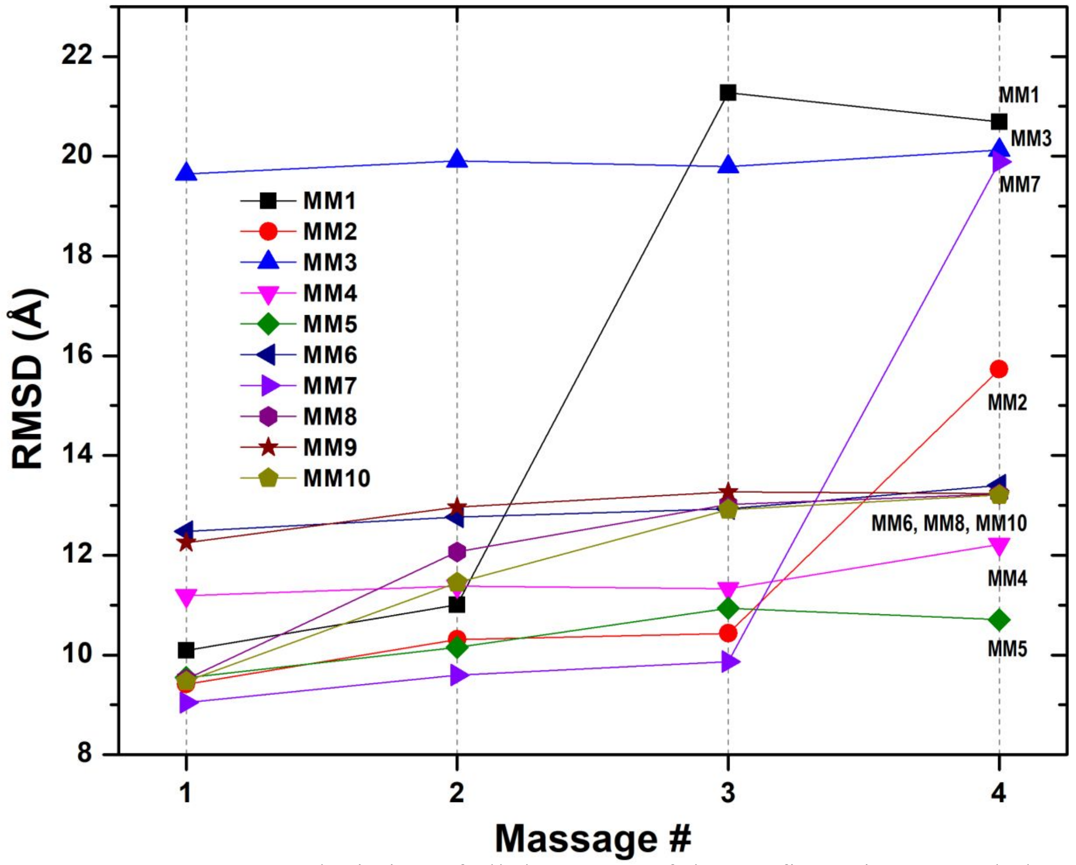

Figure S3. Root mean square deviation of all the atoms of the configurations sampled at the end of each massage (forty in all) relative to the initial structure. The initial structure is the same prototype system containing 4176 atoms (simulation box = 42 × 38 × 45 Å^3) shown in Figure S2. Each NVT MD simulation was carried out at 2000 K for 50 ps. The modified parameters are reported in **Table S2**.

Table S3. Structural descriptors of the geometries of the prototype structure obtained at the end of the *4-step-massage* and after the **GO** procedure.

MM #	C_1	C_2	C_3	C_4	$A_{(sp)}$	$A_{(sp2)}$	R_5	R_6	R_7	CN_5	sasa	D_{ave}	FV
**MM1**	0.001	0.36	0.63	0.008	10.8	2.3	0.060	0.052	0.032	34.2	10159	8.0	48.1
**opt**	0.001	0.36	0.63	0.009	6.6	2.2	0.060	0.053	0.032	33.9	9744	8.7	47.1
**MM2**	0.017	0.70	0.28	0.000	10.4	1.8	0.013	0.039	0.009	32.5	4483	26.6	42.1
**opt**	0.014	0.71	0.28	0.000	5.1	1.2	0.014	0.039	0.009	32.8	4291	23.5	41.9
**MM3**	0.001	0.32	0.67	0.004	11.2	2.1	0.054	0.061	0.039	34.0	8952	9.6	46.5
**opt**	0.000	0.32	0.68	0.004	5.2	1.6	0.054	0.062	0.039	34.0	9186	10.0	46.7
**MM4**	0.000	0.04	0.94	0.021	19.0	2.3	0.112	0.132	0.087	39.9	10329	10.1	51.2
**opt**	0.000	0.04	0.94	0.018	14.0	1.4	0.112	0.133	0.089	40.0	10118	9.5	51.5
**MM5**	0.001	0.14	0.85	0.010	14.2	2.0	0.090	0.120	0.076	38.2	10186	7.8	50.0
**opt**	0.000	0.13	0.86	0.011	8.3	1.3	0.090	0.121	0.076	38.2	10461	7.6	50.1
**MM6**	0.000	0.10	0.88	0.018	15.0	1.9	0.105	0.146	0.083	39.6	10125	11.2	50.9
**opt**	0.000	0.10	0.88	0.020	10.8	1.1	0.106	0.148	0.084	39.7	10095	11.1	51.1
**MM7**	0.003	0.96	0.04	0.000	10.8	1.4	0.000	0.000	0.000	31.1	1521	44.4	38.9
**opt**	0.001	0.93	0.07	0.000	5.1	2.7	0.000	0.000	0.000	31.6	1434	44.7	39.1
**MM8**	0.000	0.04	0.94	0.020	18.6	1.7	0.104	0.168	0.094	42.5	9812	11.8	53.0
**opt**	0.000	0.04	0.94	0.021	16.4	1.1	0.105	0.169	0.094	42.4	9929	11.5	52.7
**MM9**	0.000	0.07	0.89	0.043	17.8	1.6	0.094	0.187	0.103	43.5	10042	12.2	53.1
**opt**	0.000	0.07	0.89	0.044	13.9	1.1	0.094	0.187	0.104	43.0	9804	10.8	52.7
**MM10**	0.000	0.06	0.92	0.027	17.9	1.7	0.099	0.173	0.099	43.3	9680	10.7	52.9
**opt**	0.000	0.06	0.92	0.028	15.4	1.2	0.100	0.173	0.099	43.1	9645	12.5	52.7

D=Box density (g/cm^3)
C_1=Fraction of C atoms with coordination 1.
C_2=Fraction of C atoms with coordination 2.
C_3=Fraction of C atoms with coordination 3.
C_4=Fraction of C atoms with coordination 4.
$A_{(sp)}$=Average difference between ideal 180° angle for *sp* C atoms and effective angles formed by those C atoms characterized by coordination 2. Large values correspond to "bended wires" deviating from ideal linearity (degrees).
$A_{(sp2)}$=Average difference between the ideal 120° angle for sp^2 C atoms and effective angles formed by those C atoms characterized by coordination 3. Large values correspond to "bended sheets" deviating from perfect planarity of ideal graphitic foils (degrees).
R_5=Ratio between the number 5-member rings and the total number of atoms
R_6= Ratio between the number 6-member rings and the total number of atoms
R_7= Ratio between the number 7-member rings and the total number of atoms
CN_5=coordination number (second sphere)
sasa=Solvent-accessible surface area ($Å^2$)
D_{ave}=Average Density of the System (g/cm^3)
FV=Fraction of Free-Volume (%)

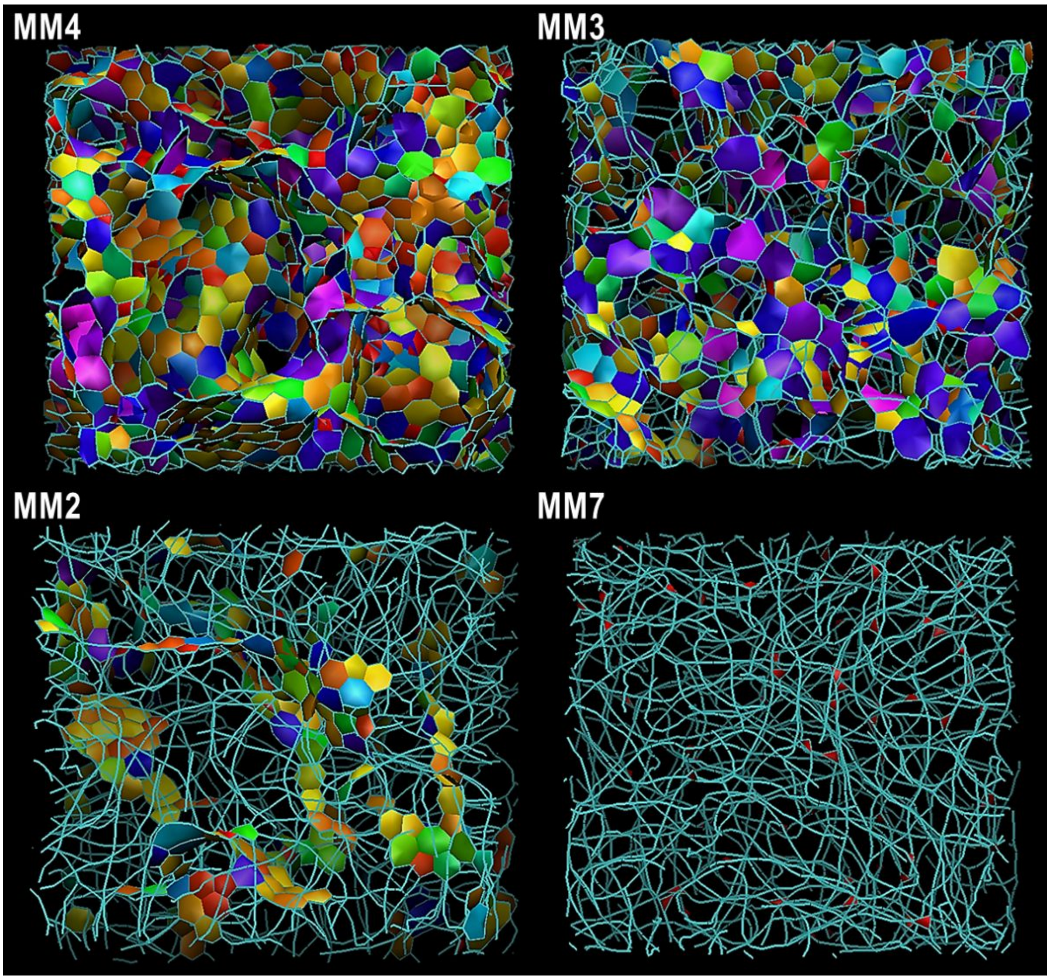

Figure S4. Final representative structures of the ten four-step-*massages* described in Tables S2-S3. The initial structure is the same prototype system containing 4176 atoms (simulation box = 42 × 38 × 45 Å^3) shown in Figure S2.

Table S4. Structural descriptors of the final geometries generated by the full DynReaxMas protocol (massages, equilibration, plus a final GO) using nine different DynReaxMas massages, namely: MM1/MM4, MM1/MM6, MM1/MM8, MM2/MM4, MM2/MM6, MM2/MM8, MM3/MM4, MM3/MM6, MM3/MM8, conducted at 2000 K and 1.15 g/cm^3 mass density (MM1/MM4 is simplified to M1M4 in the table for the sake of notation, and analogously for the other combinations).

High Density (1.15 [g/cm3]) – Equilibrated Structures – Poreblazer									
**property**	**M1M4**	**M1M6**	**M1M8**	**M2M4**	**M2M6**	**M2M8**	**M3M4**	**M3M6**	**M3M8**
density [g/cm^3]	1.146	1.146	1.146	1.146	1.146	1.146	1.146	1.146	1.146
PLD [Å]	4.74	6.94	6.90	5.99	7.36	4.62	6.02	5.83	8.24
LCD [Å]	17.89	16.36	16.93	18.81	17.46	17.94	18.89	16.88	16.48
$S_{AC,T}$ [m^2/g]	852.4	797.7	868.5	853.60	812.7	868.9	861.3	803.2	855.7
$S_{AC,A}$ [m^2/g]	602.7	644.4	742.7	723.70	729.9	646.9	713.3	572.6	643.0
$V_{PO,T}$ [cm^3/g]	0.387	0.365	0.386	0.382	0.364	0.381	0.385	0.368	0.381
$V_{PO,A}$ [cm^3/g]	0.276	0.282	0.326	0.327	0.316	0.285	0.315	0.254	0.289
$F_{He,T}$	0.44	0.42	0.44	0.44	0.42	0.44	0.44	0.42	0.44

PLD = Pore limiting diameter, Å
LCD = Largest cavity diameter, Å
$S_{AC,T}$ = Total accessible surface area, m^2/g
$S_{AC,A}$ = Network accessible surface area, m^2/g
$V_{PO,T}$ = Total probe-occupiable volume, cm^3/g
$V_{PO,A}$ = Network accessible probe-occupiable volume, cm^3/g
$F_{He,T}$ = Helium pore volume fraction

High Density (1.15 [g/cm3]) – Equilibrated Structures													
	C_1	C_2	C_3	C_4	$A_{(sp)}$	$A_{(sp2)}$	R_5	R_6	R_7	CN_5	sasa	D_{ave}	FV
**M1M4**	0.0030	0.0710	0.9000	0.0260	16.7410	1.1460	8.8840	14.7430	8.7360	41.8620	62314.2	7.4770	51.8640
**M1M6**	0.0030	0.0930	0.8910	0.0130	12.1370	1.0770	10.0260	13.3180	8.2140	40.3580	62238.8	6.6080	51.4470
**M1M8**	0.0020	0.0590	0.9210	0.0180	17.6790	1.0520	10.1410	14.5790	8.7600	41.4890	63555.6	6.9820	51.9770
**M2M4**	0.0040	0.0740	0.8960	0.0260	17.0150	1.1110	8.9200	14.7870	8.5410	41.9170	61548.0	8.8890	51.7100
**M2M6**	0.0030	0.0970	0.8870	0.0130	12.7050	1.0900	9.6980	13.1900	7.9500	40.9990	60099.2	7.2860	51.2860
**M2M8**	0.0020	0.0660	0.9130	0.0190	16.8900	1.0400	10.1490	14.5990	8.5530	40.7940	65291.5	7.0330	51.9680
**M3M4**	0.0030	0.0730	0.8960	0.0280	17.4800	1.1120	9.2390	15.0420	8.6170	41.8920	62129.2	8.0520	51.9810
**M3M6**	0.0030	0.0900	0.8930	0.0140	12.5780	1.0210	9.9340	14.0170	8.2580	40.6760	61378.3	7.2470	51.4790
**M3M8**	0.0030	0.0630	0.9170	0.0170	17.1160	1.0530	9.8780	14.7630	8.7760	41.3530	61620.0	7.1610	51.8640

C_1=Fraction of C atoms with coordination 1.
C_2=Fraction of C atoms with coordination 2.
C_3=Fraction of C atoms with coordination 3.
C_4=Fraction of C atoms with coordination 4.
$A_{(sp)}$=Average difference between ideal 180° angle for *sp* C atoms and effective angles formed by those C atoms characterized by coordination 2. Large values correspond to "bended wires" deviating from ideal linearity (degrees).
$A_{(sp2)}$=Average difference between the ideal 120° angle for sp^2 C atoms and effective angles formed by those C atoms characterized by coordination 3. Large values correspond to "bended sheets" deviating from perfect planarity of ideal graphitic foils (degrees).
R_5= 100 x Ratio between the number 5-member rings and the total number of atoms
R_6= 100 x Ratio between the number 6-member rings and the total number of atoms
R_7= 100 x Ratio between the number 7-member rings and the total number of atoms
CN_5=coordination number (second sphere)
sasa=Solvent-accessible surface area (Å^2)
D_{ave}=Average Density of the System (g/cm^3)
FV=Fraction of Free-Volume (%)

Table S5. Structural descriptors of the final geometries generated by the full DynReaxMas protocol (massages, equilibration, plus a final GO) using nine different DynReaxMas massages, namely: MM1/MM4, MM1/MM6, MM1/MM8, MM2/MM4, MM2/MM6, MM2/MM8, MM3/MM4, MM3/MM6, MM3/MM8, conducted at 2000 K and 0.50 g/cm^3 mass density (MM1/MM4 is simplified to m1m4 in the inset for the sake of notation, and analogously for the other combinations).

Medium Density (0.50 [g/cm3]) – Equilibrated Structures – Poreblazer									
property	M1M4	M1M6	M1M8	M2M4	M2M6	M2M8	M3M4	M3M6	M3M8
density [g/cm^3]	0.499	0.499	0.499	0.499	0.499	0.499	0.499	0.499	0.499
PLD [Å]	19.24	19.65	19.34	18.17	25.86	19.53	17.82	19.48	16.95
LCD [Å]	40.61	43.82	36.16	44.02	35.99	38.80	45.96	48.78	41.07
$S_{AC,T}$ [m^2/g]	2287.9	2376.8	2371.1	2221.8	2198.4	2363.5	2297.2	2258.0	2409.0
$S_{AC,A}$ [m^2/g]	2278.9	2368.5	2357.6	2213.1	2198.4	2369.9	2299.2	2245.5	2405.2
$V_{PO,T}$ [cm^3/g]	1.524	1.511	1.525	1.522	1.506	1.518	1.528	1.516	1.521
$V_{PO,A}$ [cm^3/g]	1.523	1.508	1.517	1.519	1.499	1.519	1.521	1.510	1.515
$F_{He,T}$	0.76	0.75	0.76	0.76	0.75	0.76	0.76	0.76	0.76

PLD = Pore limiting diameter, Å
LCD = Largest cavity diameter, Å
$S_{AC,T}$ = Total accessible surface area, m²/g
$S_{AC,A}$ = Network accessible surface area, m²/g
$V_{PO,T}$ = Total probe-occupiable volume, cm³/g
$V_{PO,A}$ = Network accessible probe-occupiable volume, cm³/g
$F_{He,T}$ = Helium pore volume fraction

Medium Density (0.50 [g/cm3]) – Equilibrated Structures													
	C_1	C_2	C_3	C_4	$A_{(sp)}$	$A_{(sp2)}$	R_5	R_6	R_7	CN_5	sasa	D_{ave}	FV
M1M4	0.0000	0.0960	0.8860	0.0170	20.8670	0.9530	10.5600	16.0480	9.3190	37.8590	125287.0	3.7870	78.6630
M1M6	0.0000	0.1390	0.8540	0.0070	13.7530	0.9050	10.6400	14.4120	8.0700	36.1660	131840.0	4.0210	78.2970
M1M8	0.0000	0.0970	0.8920	0.0110	19.2340	0.9760	11.1790	15.6290	8.7920	37.1110	129226.0	4.4250	78.5680
M2M4	0.0000	0.0950	0.8880	0.0170	19.5800	0.9710	10.7720	16.1080	8.4890	38.8540	121405.0	3.2940	78.6830
M2M6	0.0000	0.1240	0.8680	0.0090	13.7460	1.0620	10.6320	14.2640	8.2100	39.1720	119028.0	3.4480	78.4650
M2M8	0.0000	0.1020	0.8860	0.0120	19.2900	0.9770	10.9750	15.8840	8.4970	37.6120	127328.0	3.9390	78.5920
M3M4	0.0000	0.0960	0.8860	0.0180	19.8080	0.8880	10.2890	16.3190	8.7480	38.4100	123729.0	3.7030	78.7540
M3M6	0.0000	0.1180	0.8720	0.0090	13.7950	0.8760	10.6760	15.2860	8.1460	37.7050	123707.0	3.6960	78.6100
M3M8	0.0000	0.1040	0.8840	0.0120	18.8810	0.8770	10.8960	16.1040	8.6450	37.2830	130090.0	3.3690	78.6540

C_1=Fraction of C atoms with coordination 1.
C_2=Fraction of C atoms with coordination 2.
C_3=Fraction of C atoms with coordination 3.
C_4=Fraction of C atoms with coordination 4.
$A_{(sp)}$=Average difference between ideal 180° angle for *sp* C atoms and effective angles formed by those C atoms characterized by coordination 2. Large values correspond to "bended wires" deviating from ideal linearity (degrees).
$A_{(sp2)}$=Average difference between the ideal 120° angle for sp^2 C atoms and effective angles formed by those C atoms characterized by coordination 3. Large values correspond to "bended sheets" deviating from perfect planarity of ideal graphitic foils (degrees).
R_5= 100 x Ratio between the number 5-member rings and the total number of atoms
R_6= 100 x Ratio between the number 6-member rings and the total number of atoms
R_7= 100 x Ratio between the number 7-member rings and the total number of atoms
CN_5=coordination number (second sphere)
sasa=Solvent-accessible surface area (Å^2)
D_{ave}=Average Density of the System (g/cm^3)
FV=Fraction of Free-Volume (%)

Table S6. Structural descriptors of the final geometries generated by the full DynReaxMas protocol (massages, equilibration, plus a final GO) using nine different DynReaxMas massages, namely: MM1/MM4, MM1/MM6, MM1/MM8, MM2/MM4, MM2/MM6, MM2/MM8, MM3/MM4, MM3/MM6, MM3/MM8, conducted at 2000 K and 0.16 g/cm^3 mass density (MM1/MM4 is simplified to m1m4 in the inset for the sake of notation, and analogously for the other combinations).

Low Density (0.16 [g/cm3]) – Equilibrated Structures – Poreblazer									
**property**	**M1M4**	**M1M6**	**M1M8**	**M2M4**	**M2M6**	**M2M8**	**M3M4**	**M3M6**	**M3M8**
density [g/cm^3]	0.164	0.164	0.164	0.164	0.164	0.164	0.164	0.164	0.164
PLD [Å]	111.82	114.66	109.94	57.61	78.46	105.71	54.60	46.01	48.50
LCD [Å]	135.93	128.27	134.34	106.07	99.82	119.66	97.51	92.09	91.48
$S_{AC,T}$ [m^2/g]	1554.3	1718.9	1620.8	2193.1	1282.2	2023.2	3511.9	3604.2	3635.0
$S_{AC,A}$ [m^2/g]	1530.3	1705.4	1607.6	2178.9	1259.4	1991.0	3513.3	3590.2	3633.8
$V_{PO,T}$ [cm^3/g]	5.64	5.62	5.64	5.62	5.61	5.63	5.62	5.62	5.62
$V_{PO,A}$ [cm^3/g]	5.62	5.61	5.62	5.62	5.59	5.62	5.62	5.62	5.62
$F_{He,T}$	0.92	0.92	0.92	0.92	0.92	0.92	0.92	0.92	0.92

PLD = Pore limiting diameter, Å
LCD = Largest cavity diameter, Å
$S_{AC,T}$ = Total accessible surface area, m^2/g
$S_{AC,A}$ = Network accessible surface area, m^2/g
$V_{PO,T}$ = Total probe-occupiable volume, cm^3/g
$V_{PO,A}$ = Network accessible probe-occupiable volume, cm^3/g
$F_{He,T}$ = Helium pore volume fraction

Low Density (0.16 [g/cm3]) – Equilibrated Structures													
	C_1	C_2	C_3	C_4	$A_{(sp)}$	$A_{(sp2)}$	R_5	R_6	R_7	CN_5	sasa	D_{ave}	FV
**M1M4**	0.0000	0.0560	0.9140	0.0300	17.3220	1.1770	9.9100	16.0920	8.9320	55.5100	84098.6	2.9370	93.2320
**M1M6**	0.0000	0.0790	0.9070	0.0150	13.2810	1.0770	10.8840	14.7430	8.5610	52.1660	91223.8	3.4090	93.1150
**M1M8**	0.0000	0.0570	0.9250	0.0180	16.7730	1.1310	10.6680	15.9080	8.7680	54.8360	85578.6	3.6680	93.1930
**M2M4**	0.0000	0.0900	0.8840	0.0250	19.3410	1.1330	9.8940	15.9280	8.5930	51.4260	111729.	4.4020	93.0670
**M2M6**	0.0010	0.0650	0.9180	0.0150	13.4370	1.2380	10.4250	14.3600	8.2020	57.7050	68929.0	4.7610	93.1880
**M2M8**	0.0000	0.0790	0.9030	0.0180	16.9770	1.1910	10.5760	15.6890	8.3730	52.4630	102666	3.5440	93.1260
**M3M4**	0.0000	0.1390	0.8500	0.0110	21.5580	0.8290	11.0070	15.6850	8.2930	37.6690	171616	2.8300	92.8330
**M3M6**	0.0000	0.1580	0.8380	0.0040	16.0130	0.7640	10.9630	14.8590	7.7510	36.2800	175740	2.7230	92.7950
**M3M8**	0.0000	0.1410	0.8540	0.0060	19.9620	0.8430	11.2070	15.4450	7.9780	36.6920	177079	3.1170	92.8290

C_1=Fraction of C atoms with coordination 1.
C_2=Fraction of C atoms with coordination 2.
C_3=Fraction of C atoms with coordination 3.
C_4=Fraction of C atoms with coordination 4.
$A_{(sp)}$=Average difference between ideal 180° angle for *sp* C atoms and effective angles formed by those C atoms characterized by coordination 2. Large values correspond to “bended wires” deviating from ideal linearity (degrees).
$A_{(sp2)}$=Average difference between the ideal 120° angle for *sp*^{2} C atoms and effective angles formed by those C atoms characterized by coordination 3. Large values correspond to “bended sheets” deviating from perfect planarity of ideal graphitic foils (degrees).
R_5= 100 x Ratio between the number 5-member rings and the total number of atoms
R_6= 100 x Ratio between the number 6-member rings and the total number of atoms
R_7= 100 x Ratio between the number 7-member rings and the total number of atoms
CN_5=coordination number (second sphere)
sasa=Solvent-accessible surface area (Å^2)
D_{ave}=Average Density of the System (g/cm^3)
FV=Fraction of Free-Volume (%)

Convergence Check

For the three examined densities (1.15, 0.50, and 0.16 g/cm^3) and for all combinations of massages {MM1,MM2,MM3} x {MM4,MM6,MM8}, the final structure of the last massage was equilibrated for about fifty picoseconds (ps) using the original (unbiased, not-massaged) ReaxFF (C.ff) force field at T=2000 K. The average configuration obtained from the final ten ps of this equilibration was used for the subsequent GO steps whence the final descriptor analysis. To check convergence, the root mean squared deviations (RMSD) of all the atoms from the average structure were evaluated in the last ten ps of the equilibration dynamics: these are illustrated in **Figure S5(left panel)** focusing on the intermediate density (0.50 g/cm^3). A superimposition of the average structure with another structure having a RMSD of approximately 7.0 Å obtained during the last ten ps of the dynamics is also shown in **Figure S5(right panel)**. Inspection of **Figure S5** indicates that the RMSD are small and oscillate in a range of 6.2-8.5 Å. Visual inspection of the superimposition further confirms the validity of convergence.

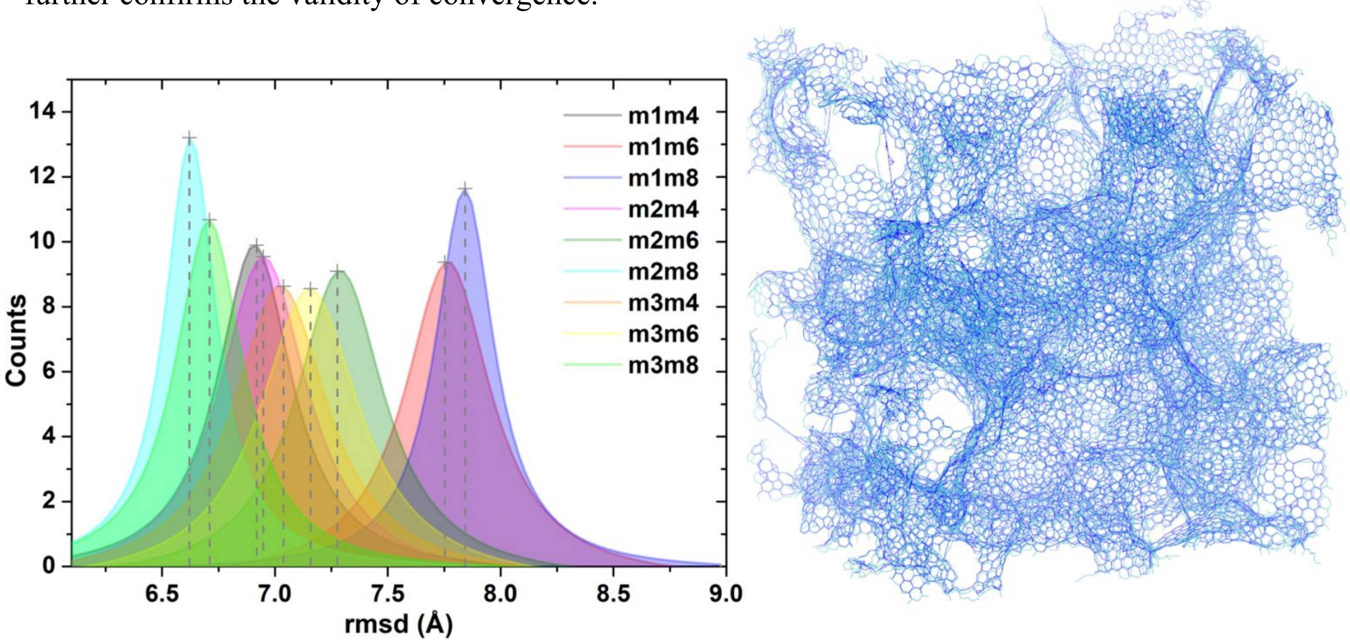

Figure S5. On the left, distribution of the RMSD of all the structures sampled during the last ten ps of the equilibration dynamics with the C.ff force field at T=2000K relative to the average configuration. Results refer to mass density of 0.50 g/cm^3 and are reported for all combinations of massages: {MM1,MM2,MM3} x {MM4,MM6,MM8} (MM1/MM4 is simplified to m1m4 in the inset for the sake of notation, and analogously for the other combinations). On the right, a typical superimposition between all the atoms of the average structure and those of a configuration sampled during the last ten picoseconds of the equilibration dynamics having a RMSD=7 Å.

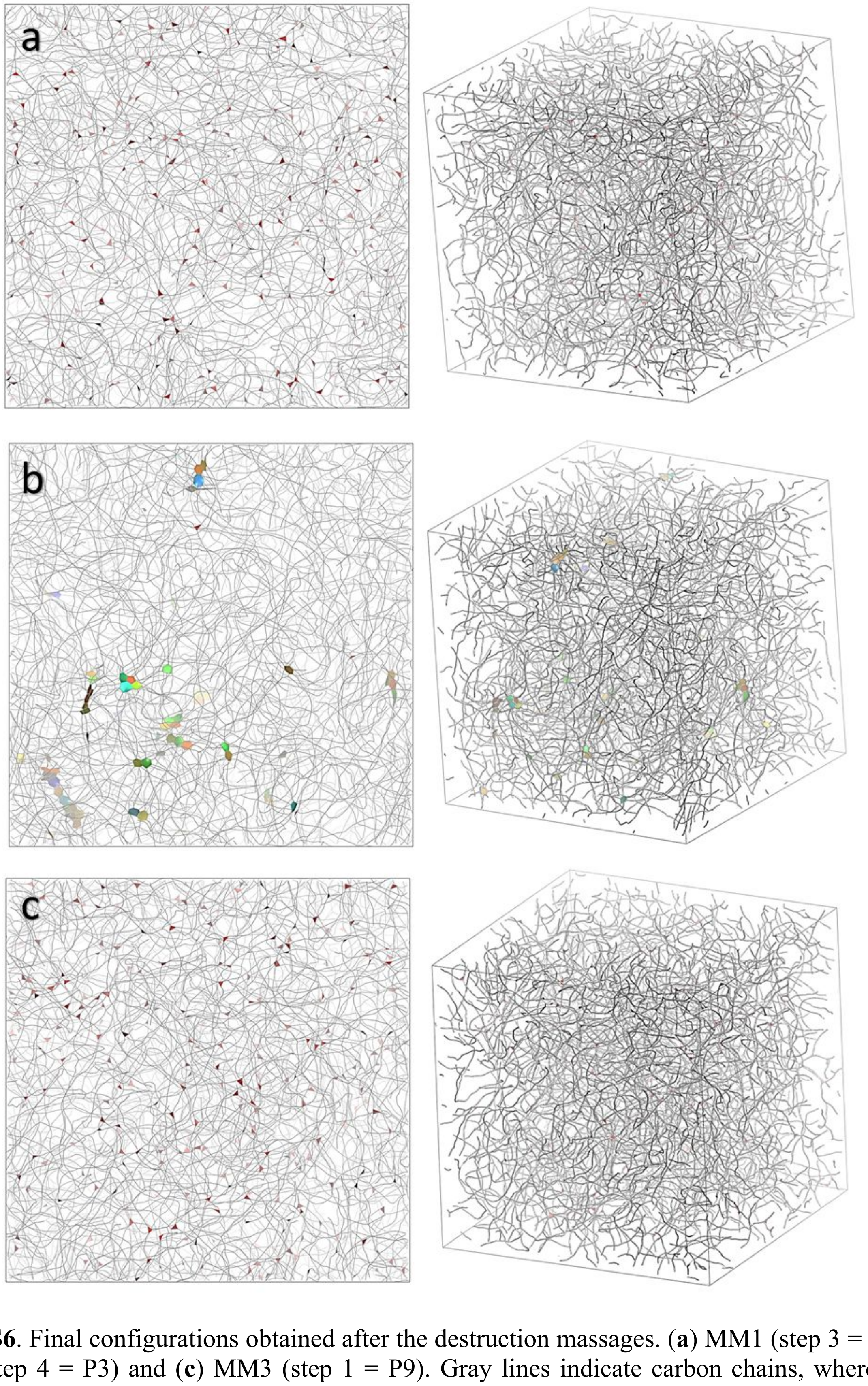

Figure S6. Final configurations obtained after the destruction massages. (**a**) MM1 (step 3 = P9), (**b**) MM2 (step 4 = P3) and (**c**) MM3 (step 1 = P9). Gray lines indicate carbon chains, whereas ring present in the configurations are identified by multicolored regions (3-member rings are red).

Effects of the temperature selected for the DynReaxMas massage procedure

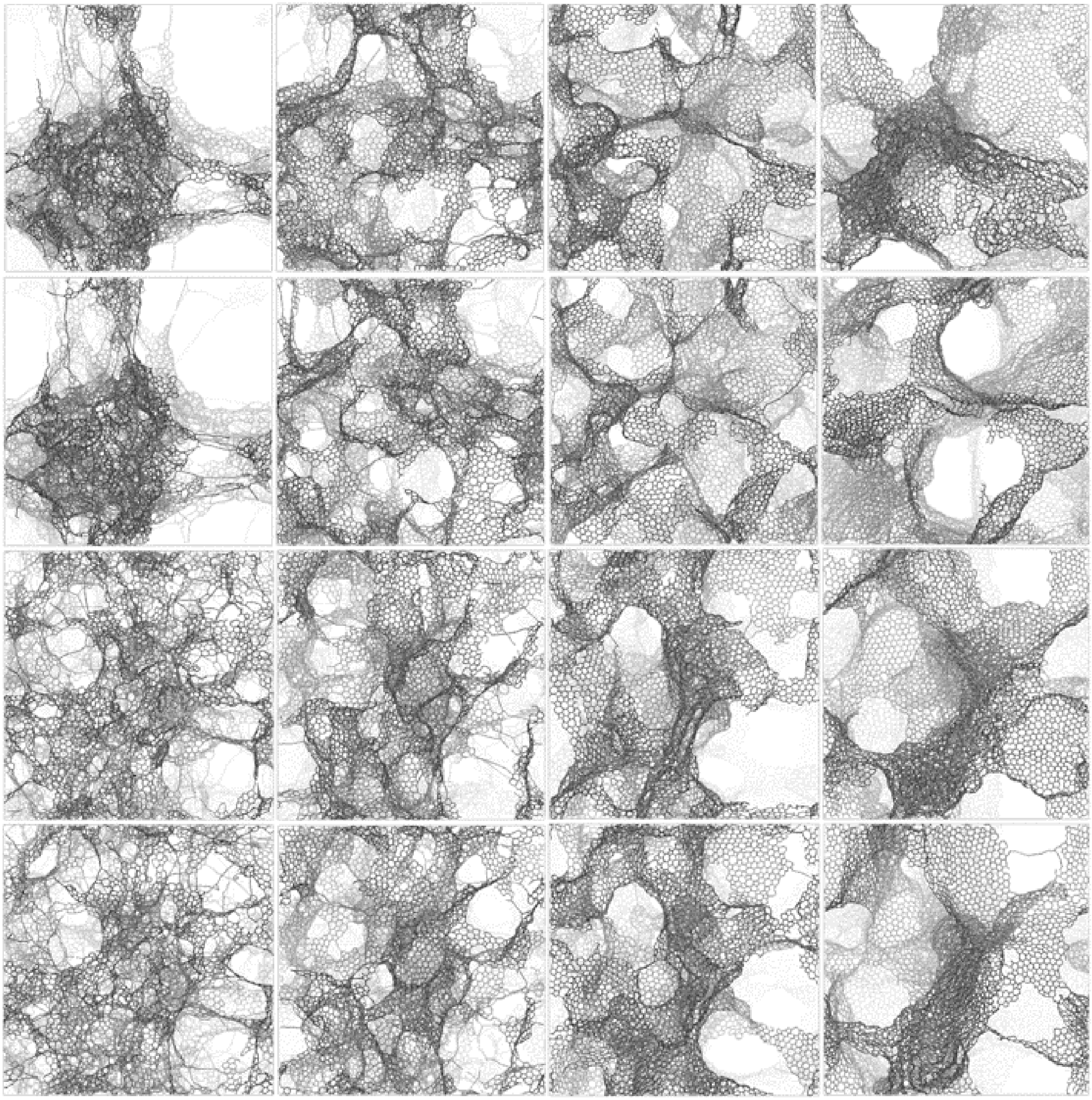

Figure S7. Final configurations obtained after four different DynReaxMas massages, namely: MM1/MM4, MM1/MM8, MM3/MM4, MM3/MM8 (from top to bottom), at four different temperatures, namely: 1500, 2000, 2500, and 3000K (from left to right).

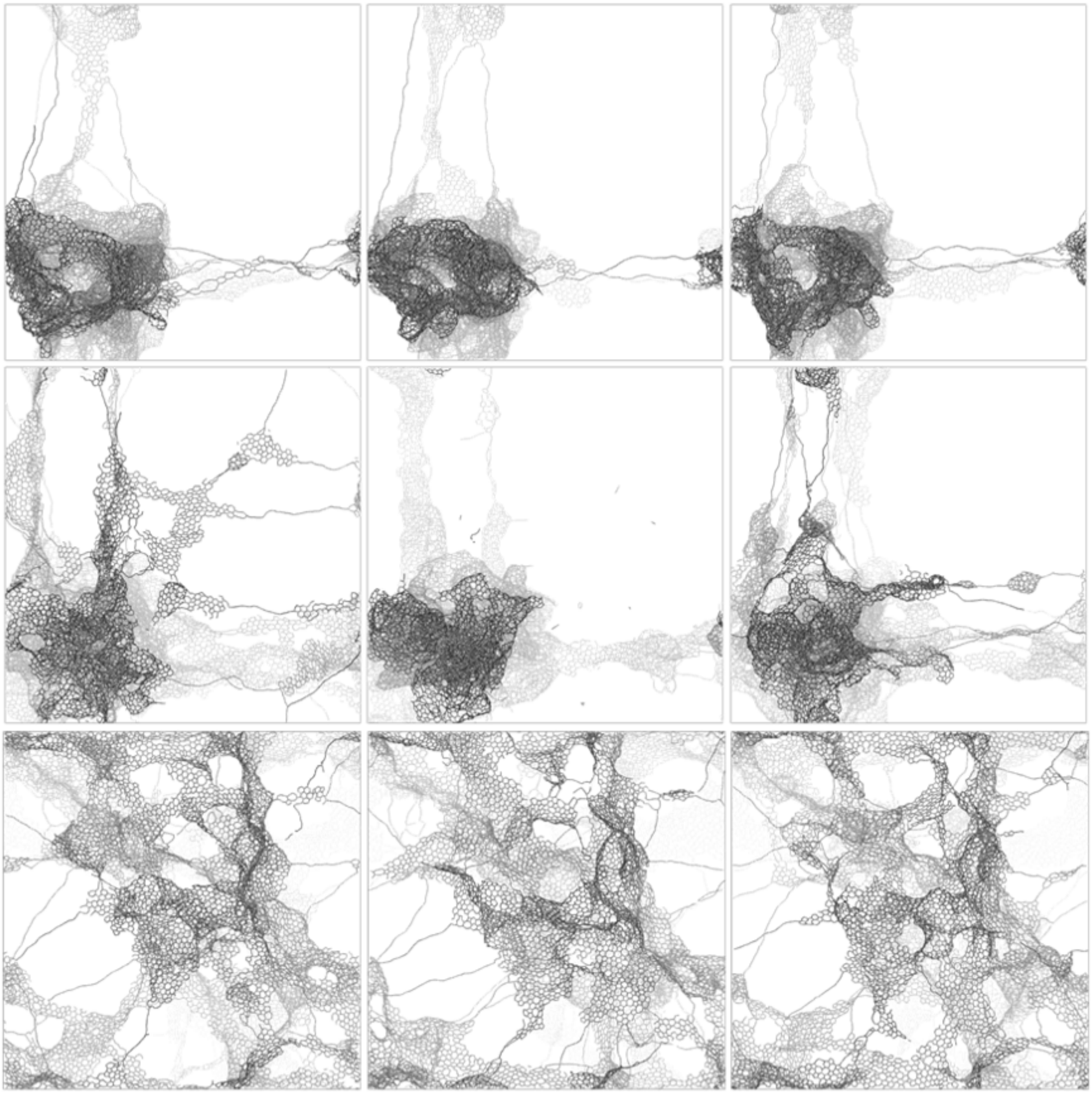

Figure S8. Final configurations (at LOW density – 0.16 g/cm^3) obtained after nine different combinations of massages, namely: MM1/MM4, MM1/MM6, MM1/MM8 (first row), MM2/MM4, MM2/MM6, MM2/MM8 (second row), MM3/MM4, MM3/MM6, MM3/MM8 (third row).

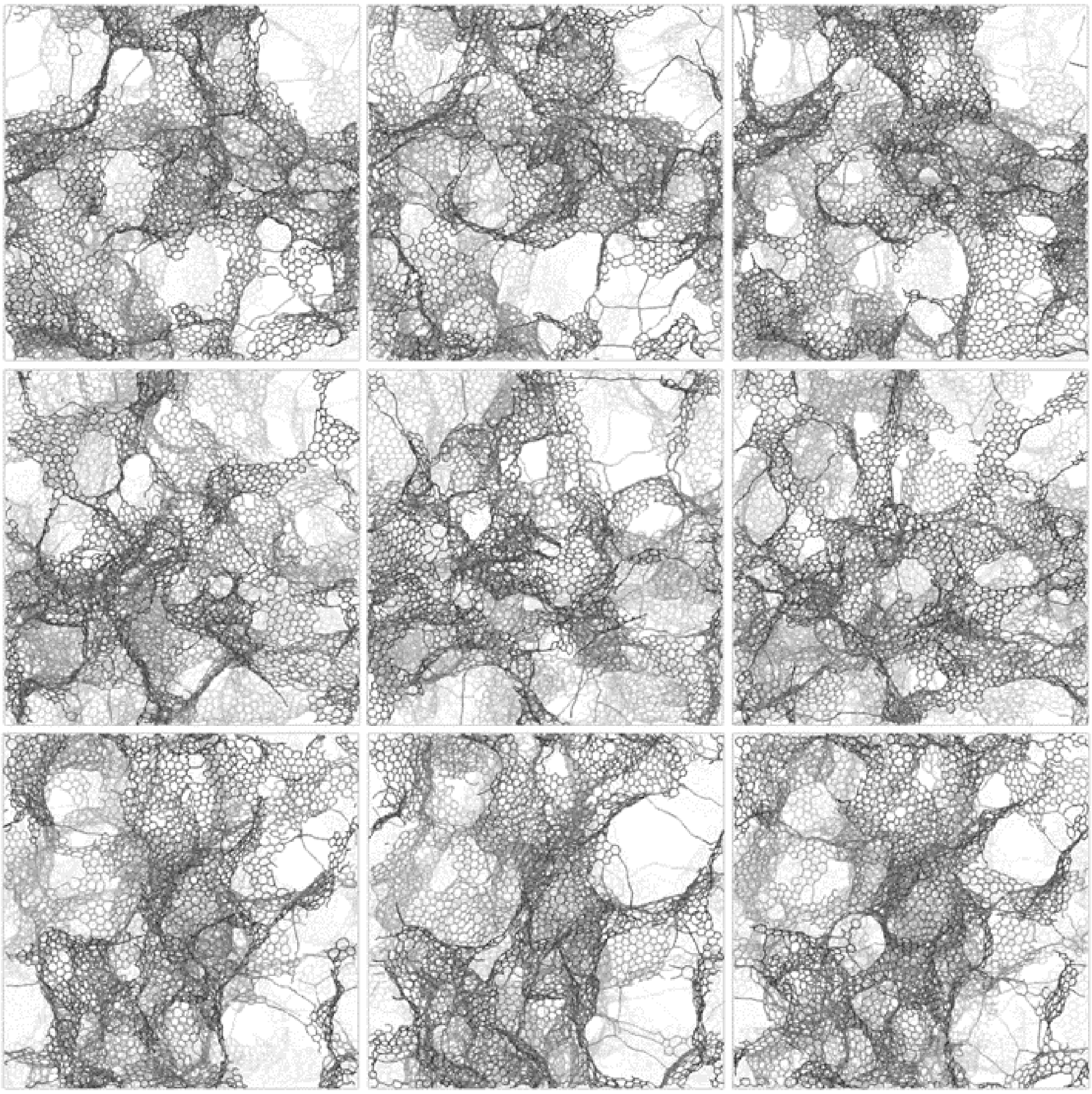

Figure S9. Final configurations (at MEDIUM density – 0.50 g/cm^3) obtained after nine different combinations of massages, namely: MM1/MM4, MM1/MM6, MM1/MM8 (first row), MM2/MM4, MM2/MM6, MM2/MM8 (second row), MM3/MM4, MM3/MM6, MM3/MM8 (third row).

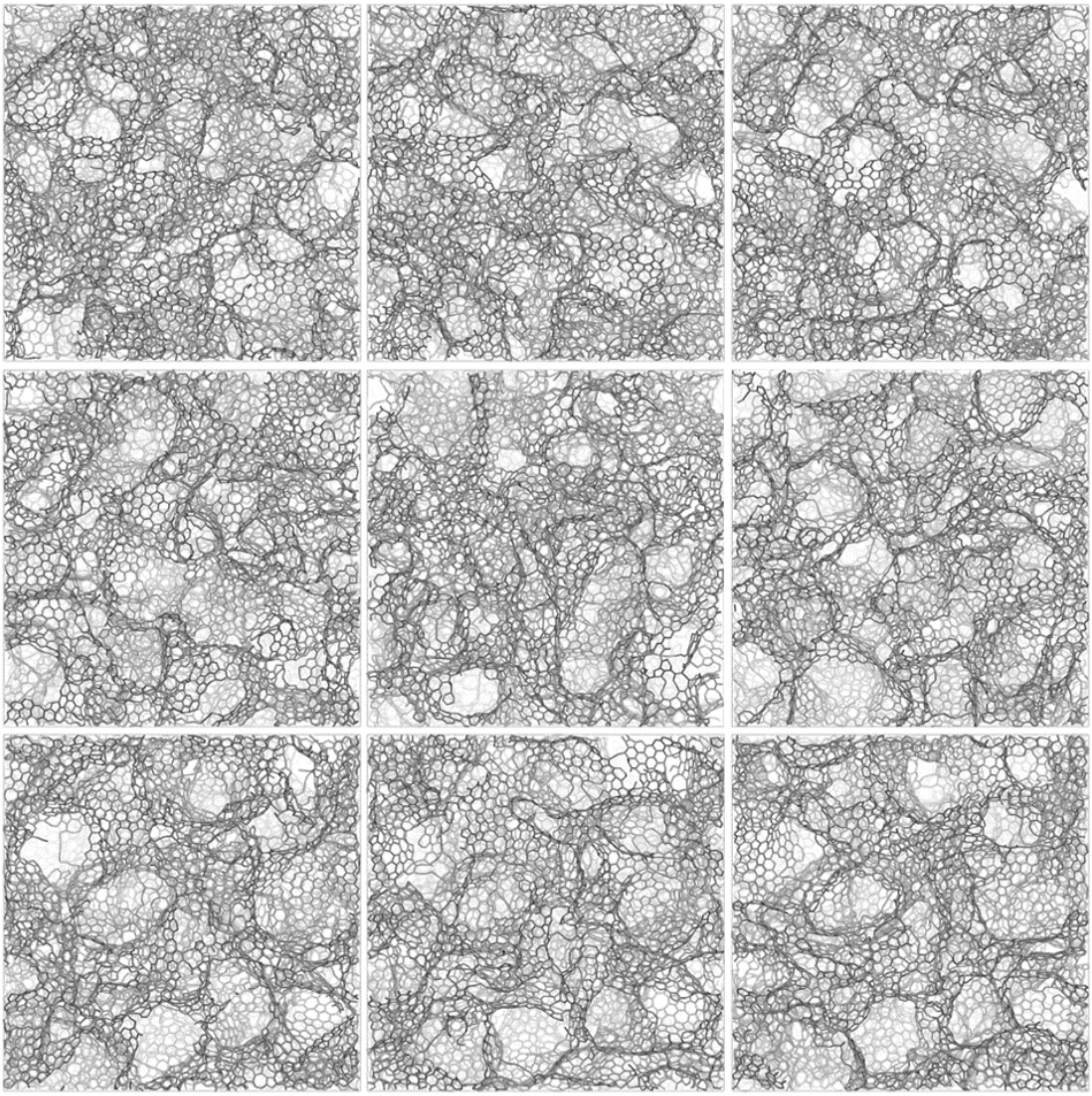

Figure S10. Final configurations (at HIGH density – 1.15 g/cm^3) obtained after nine different combinations of massages, namely: MM1/MM4, MM1/MM6, MM1/MM8 (first row), MM2/MM4, MM2/MM6, MM2/MM8 (second row), MM3/MM4, MM3/MM6, MM3/MM8 (third row).

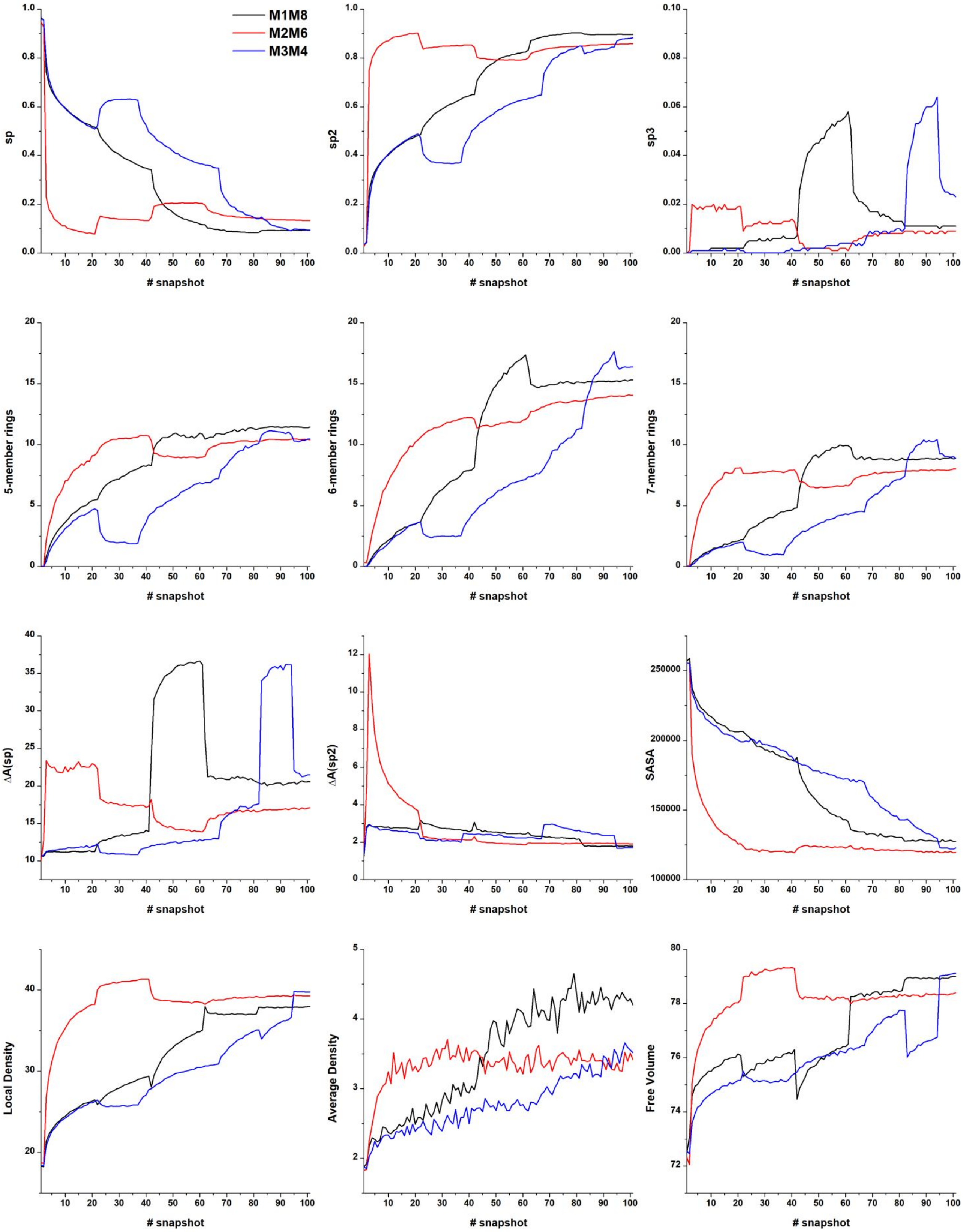

Figure S11. Evolution of the given descriptors [sp = C_2, sp2 = C_3, sp3 = C_4, 5-member rings = R_5, 6-member rings = R_6, 7-member rings = R_7, $\Delta A_{(sp)}$, $\Delta A_{(sp2)}$, SASA, Local Density, Average Density = D_{ave}, Free Volume = FV, as defined in Tables S3 and S4) during the various massages after destruction for MM1/MM8, MM2/MM6, and MM3/MM4 DynReaxMas runs at medium density (0.50 g/cm^3) and 2000K (MM1/MM4 is simplified to m1m4 in the inset, and analogously for the other combinations). Twenty points are reported from each parameter perturbation.

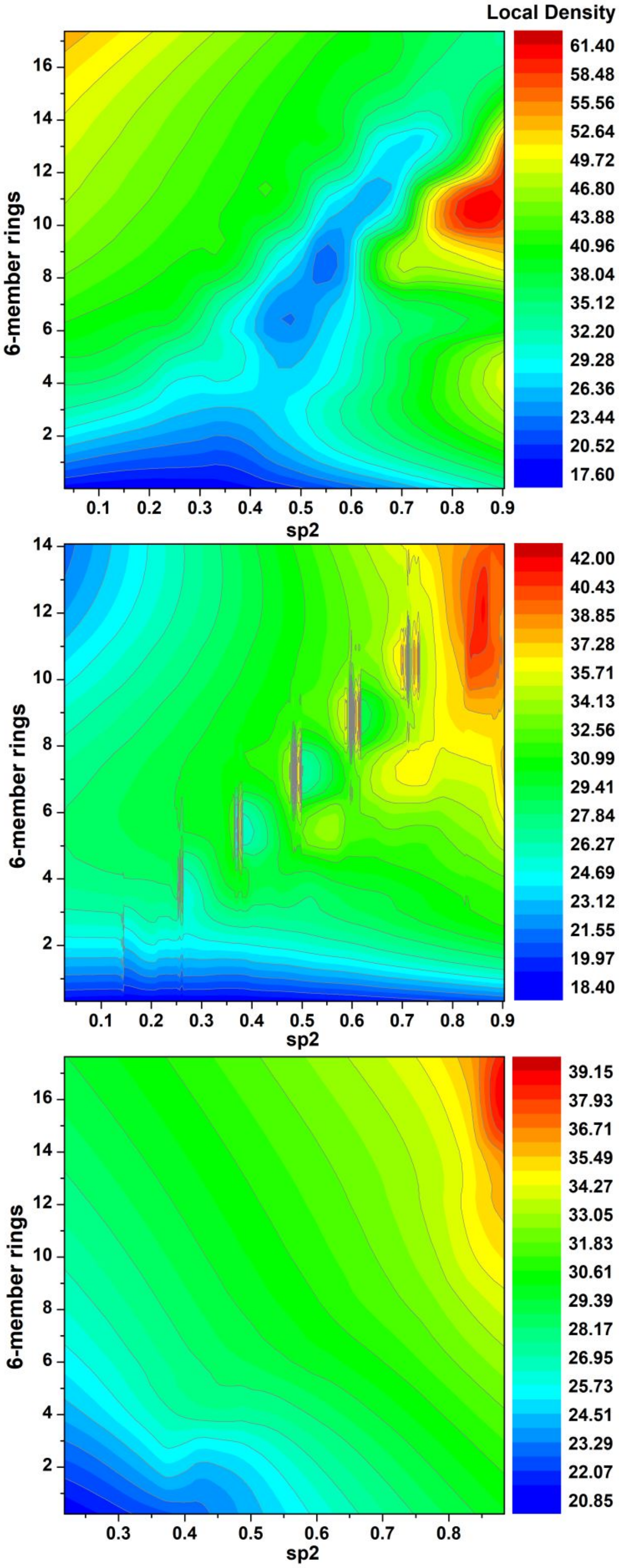

Figure S12. Contour plots (derived from the parameters evolution) highlighting the correlation among sp2 (or C_3 in Tables S3 and S4), 6-member ring content (R_6 in the Tables), and local density (multicolored regions) for MM1/MM8 (top), MM2/MM6 (medium), and MM3/MM4 (bottom) DynReaxMas runs, at medium-density (0.50 g/cm^3) and 2000K.

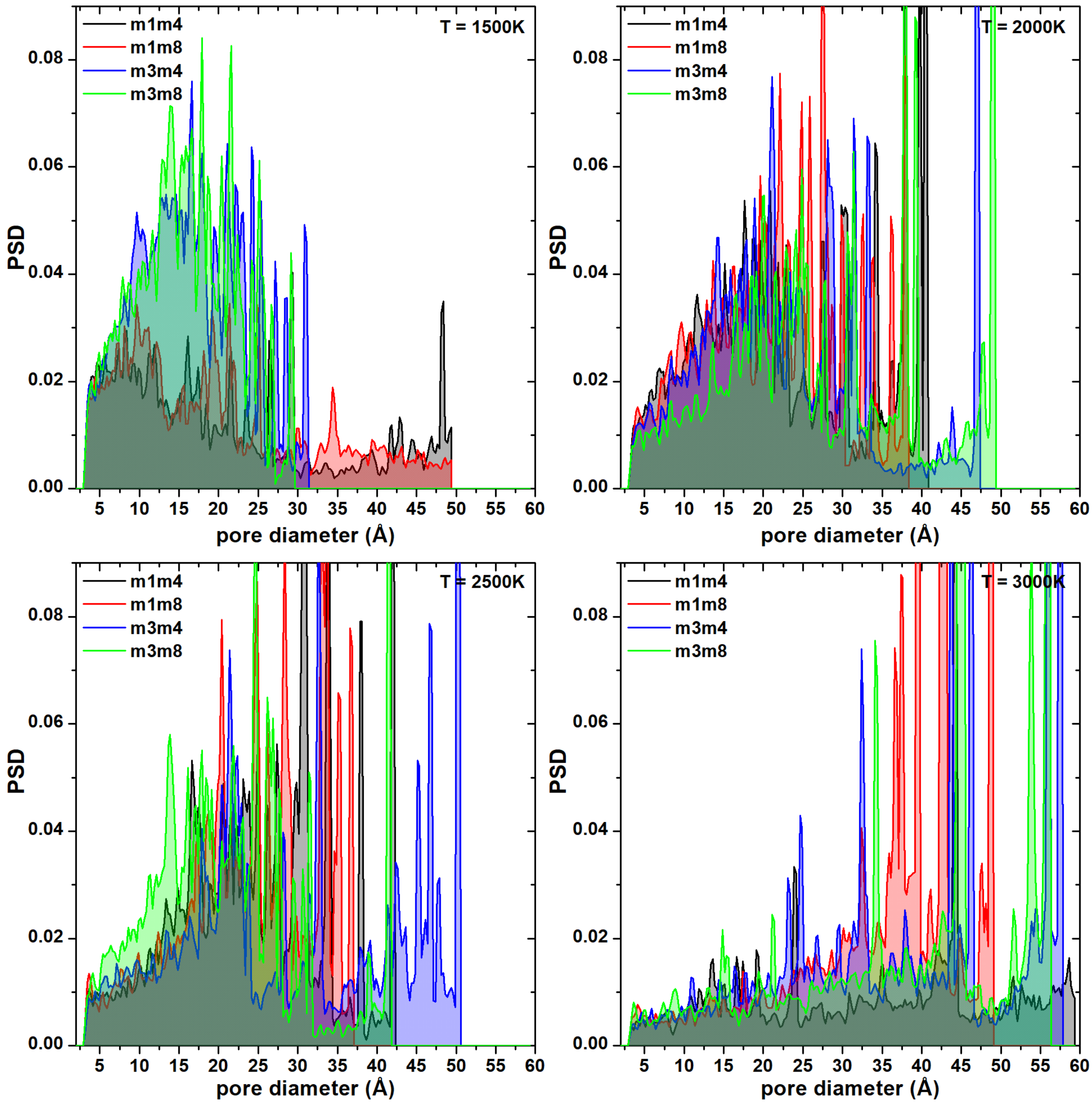

Figure S13. Pore size distributions of the final structures deriving from four different DynReaxMas massages, namely: MM1/MM4, MM1/MM8, MM3/MM4, MM3/MM8, (MM1/MM4 is simplified to m1m4 in the inset for the sake of notation, and analogously for the other combinations) conducted at four different temperatures, namely: 1500, 2000, 2500, and 3000 K and at medium-density (0.50 g/cm^3).

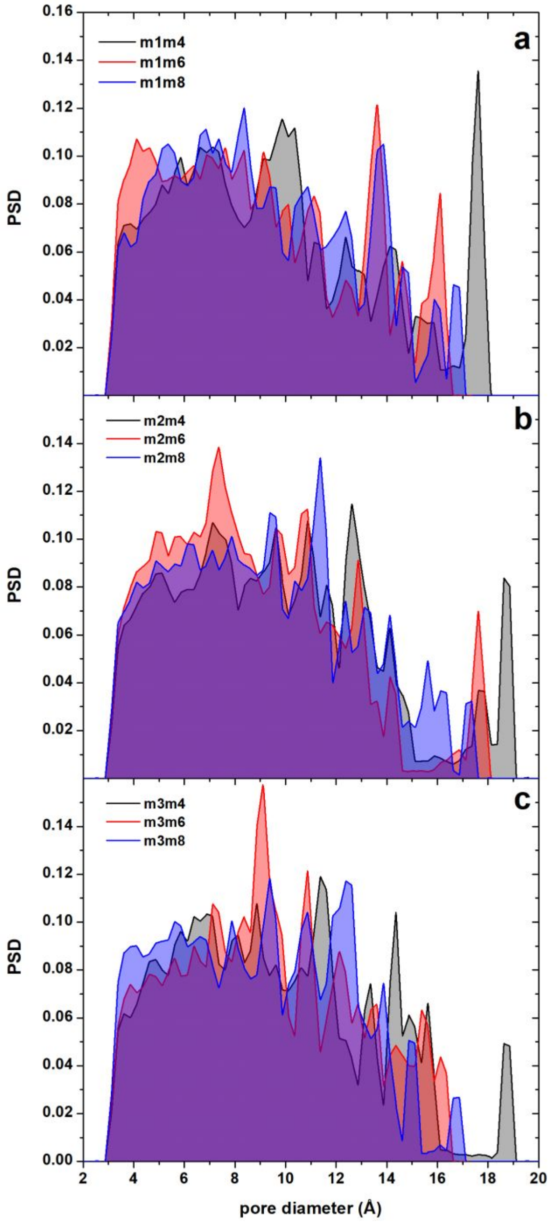

Figure S14. Pore size distributions (PSD) of the final structures deriving from nine different DynReaxMas massages, namely: MM1/MM4, MM1/MM6, MM1/MM8 (top), MM2/MM4, MM2/MM6, MM2/MM8 (middle), MM3/MM4, MM3/MM6, MM3/MM8 (bottom), conducted at 2000 K and 1.15 g/cm^3 mass density (MM1/MM4 is simplified to m1m4 in the inset for the sake of notation, and analogously for the other combinations).

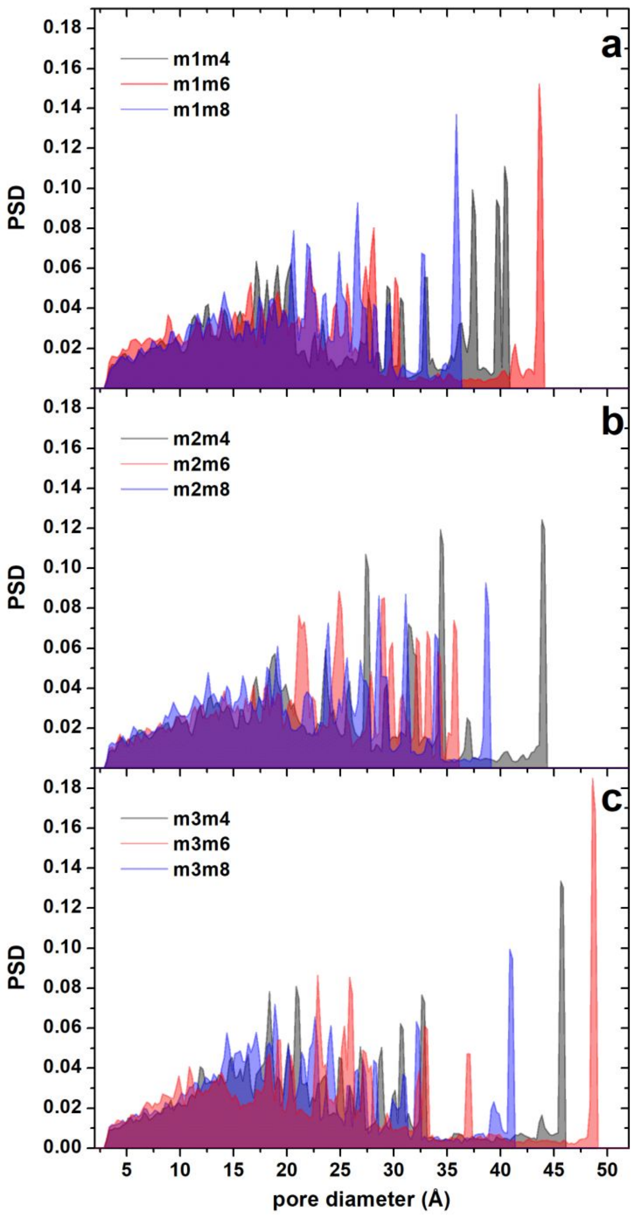

Figure S15. Pore size distributions (PSD) of the final structures deriving from nine different DynReaxMas massages, namely: MM1/MM4, MM1/MM6, MM1/MM8 (top), MM2/MM4, MM2/MM6, MM2/MM8 (middle), MM3/MM4, MM3/MM6, MM3/MM8 (bottom), conducted at 2000 K and 0.50 g/cm^3 mass density (MM1/MM4 is simplified to m1m4 in the inset for the sake of notation, and analogously for the other combinations).

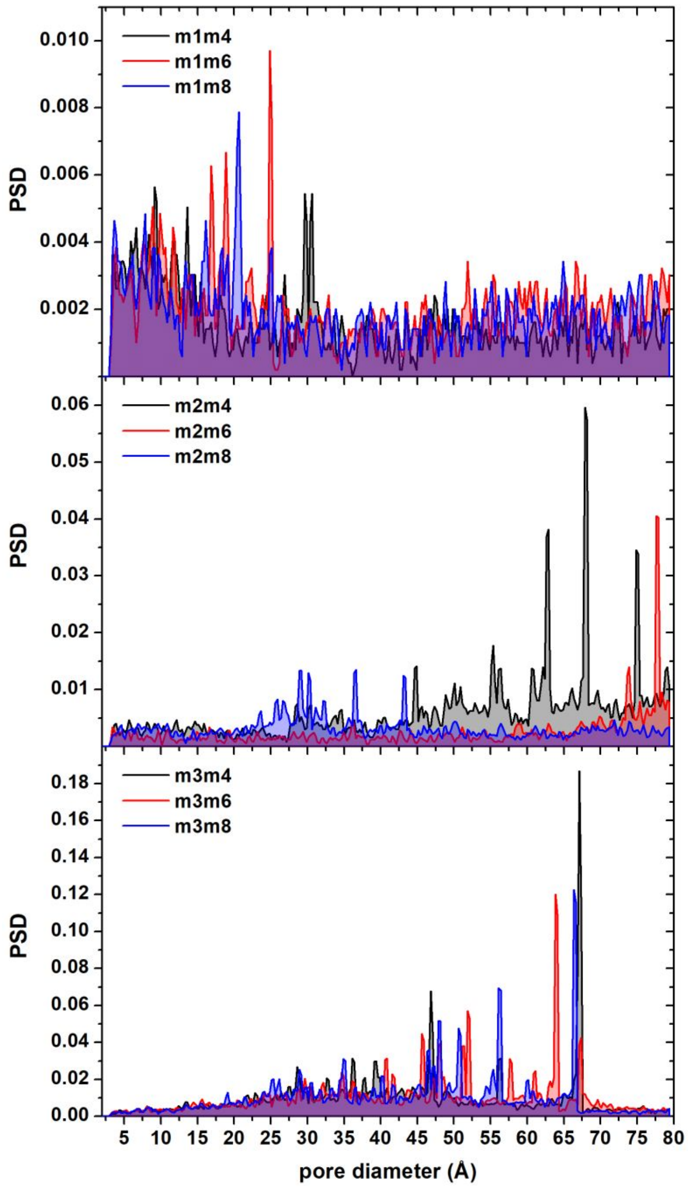

Figure S16. Pore size distributions (PSD) of the final structures deriving from nine different DynReaxMas massages, namely: MM1/MM4, MM1/MM6, MM1/MM8 (top), MM2/MM4, MM2/MM6, MM2/MM8 (middle), MM3/MM4, MM3/MM6, MM3/MM8 (bottom), conducted at 2000 K and 0.16 g/cm^3 mass density (MM1/MM4 is simplified to m1m4 in the inset for the sake of notation, and analogously for the other combinations).

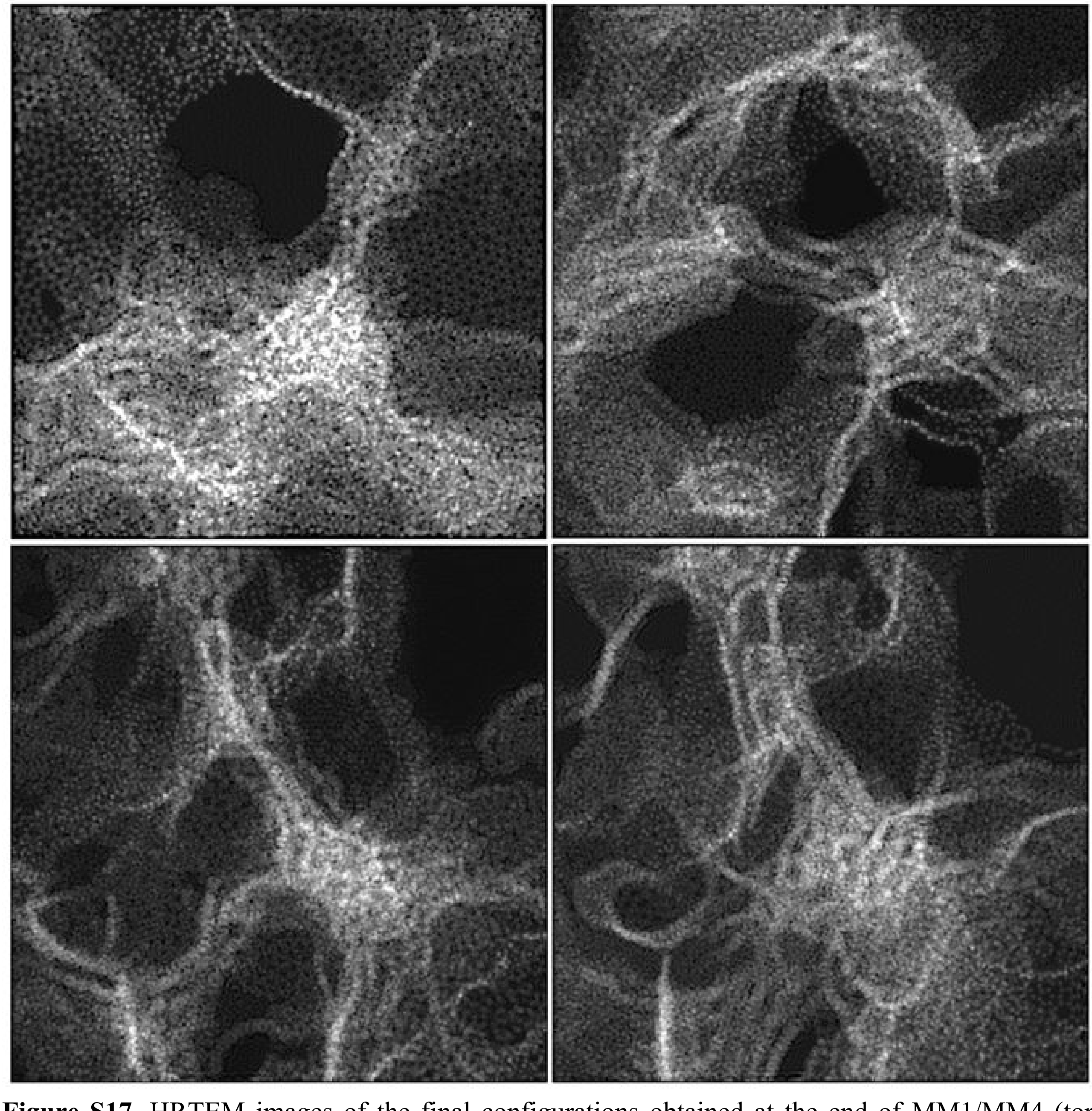

Figure S17. HRTEM images of the final configurations obtained at the end of MM1/MM4 (top left), MM1/MM8 (top right), MM3/MM4 (bottom left), MM3/MM8 (bottom right) at medium-density (0.50 g/cm^3) and T=3000K.

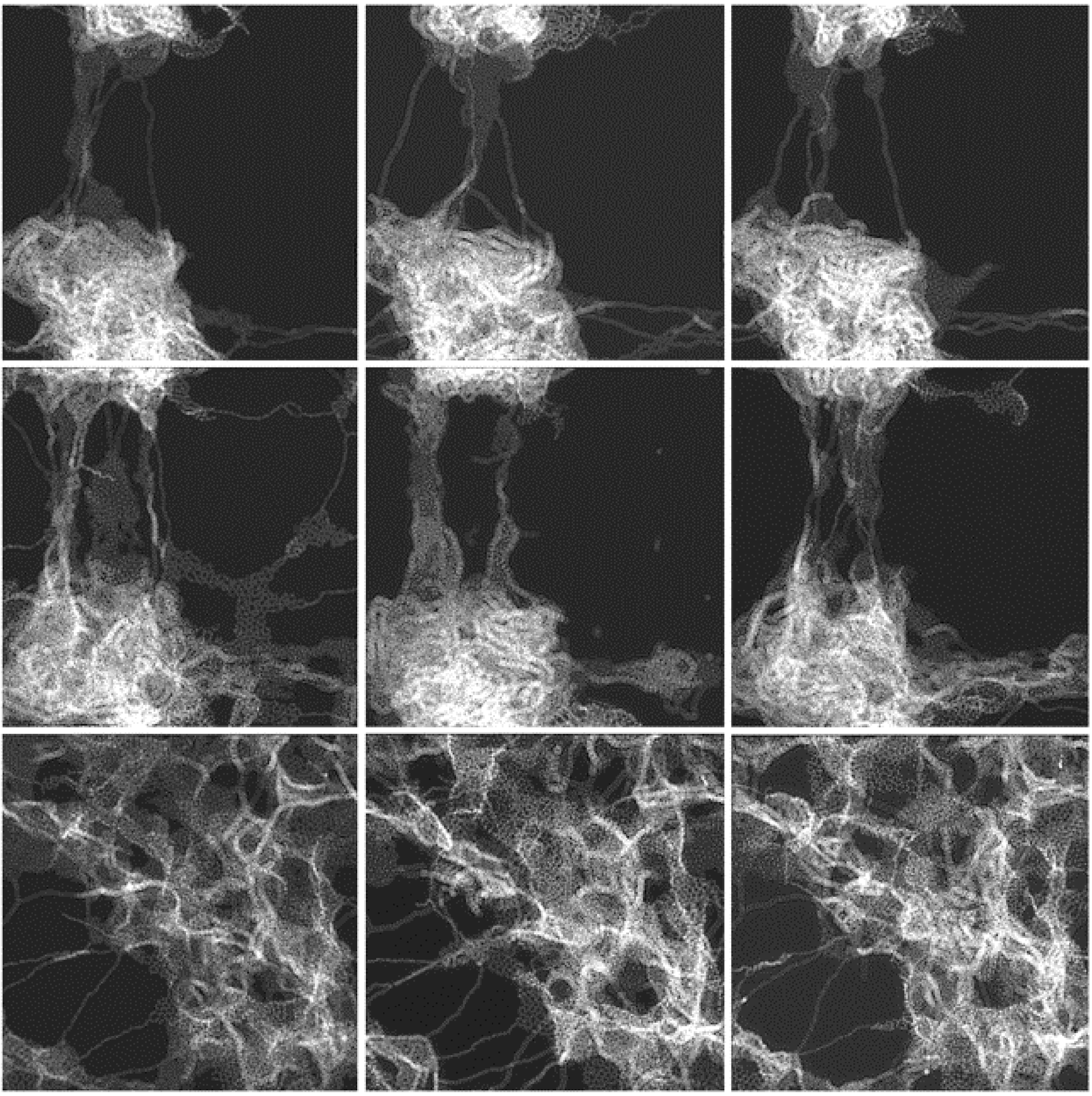

Figure S18. HRTEM images of the final configurations (at LOW density – 0.16 g/cm^3, T = 2000 K) obtained after nine different massages, namely: MM1/MM4, MM1/MM6, MM1/MM8 (first row), MM2/MM4, MM2/MM6, MM2/MM8 (second row), MM3/MM4, MM3/MM6, MM3/MM8 (third row).

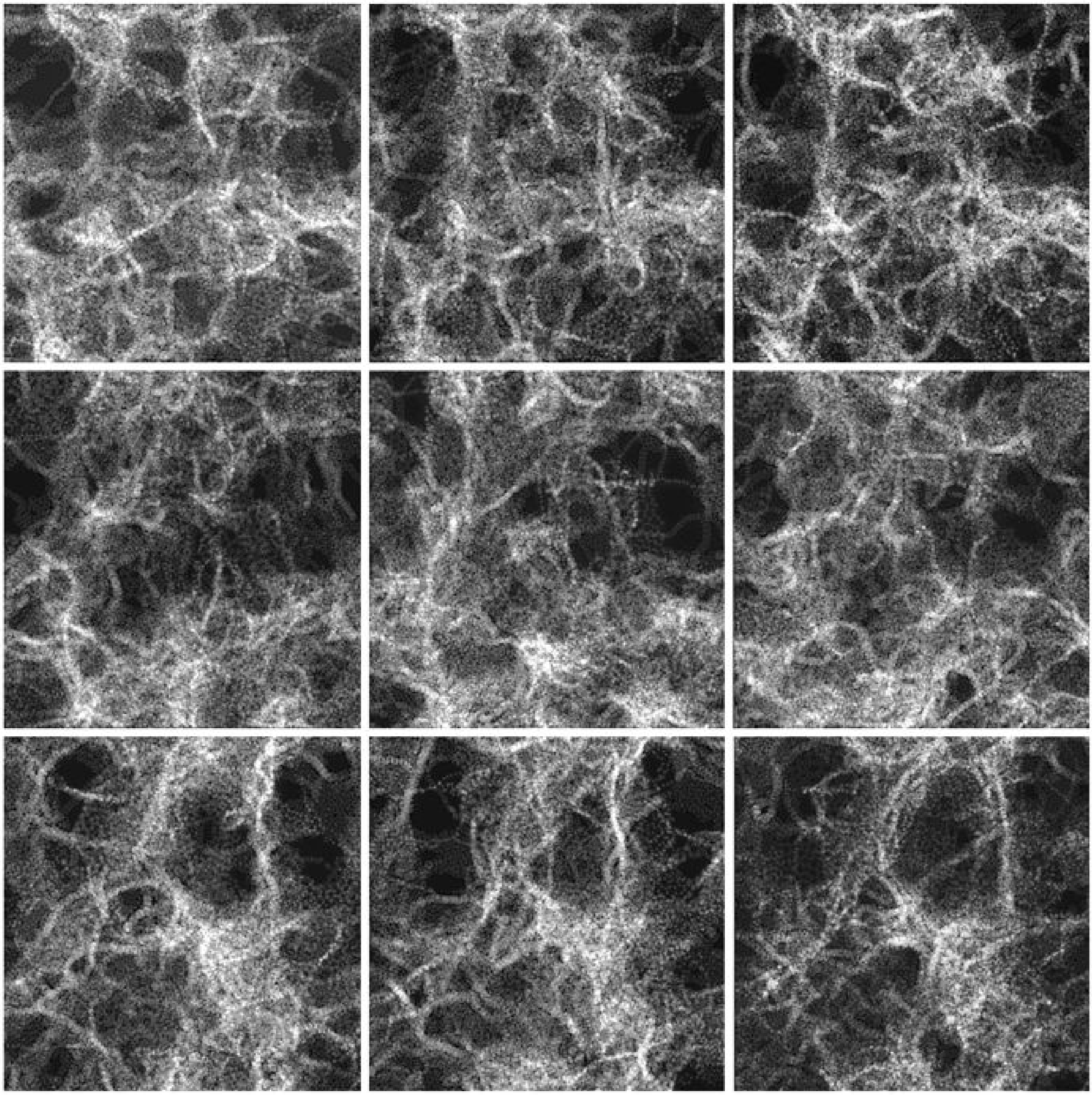

Figure S19. HRTEM images of the final configurations (at MEDIUM density – 0.50 g/cm^3, T = 2000 K) obtained after nine different massages, namely: MM1/MM4, MM1/MM6, MM1/MM8 (first row), MM2/MM4, MM2/MM6, MM2/MM8 (second row), MM3/MM4, MM3/MM6, MM3/MM8 (third row).

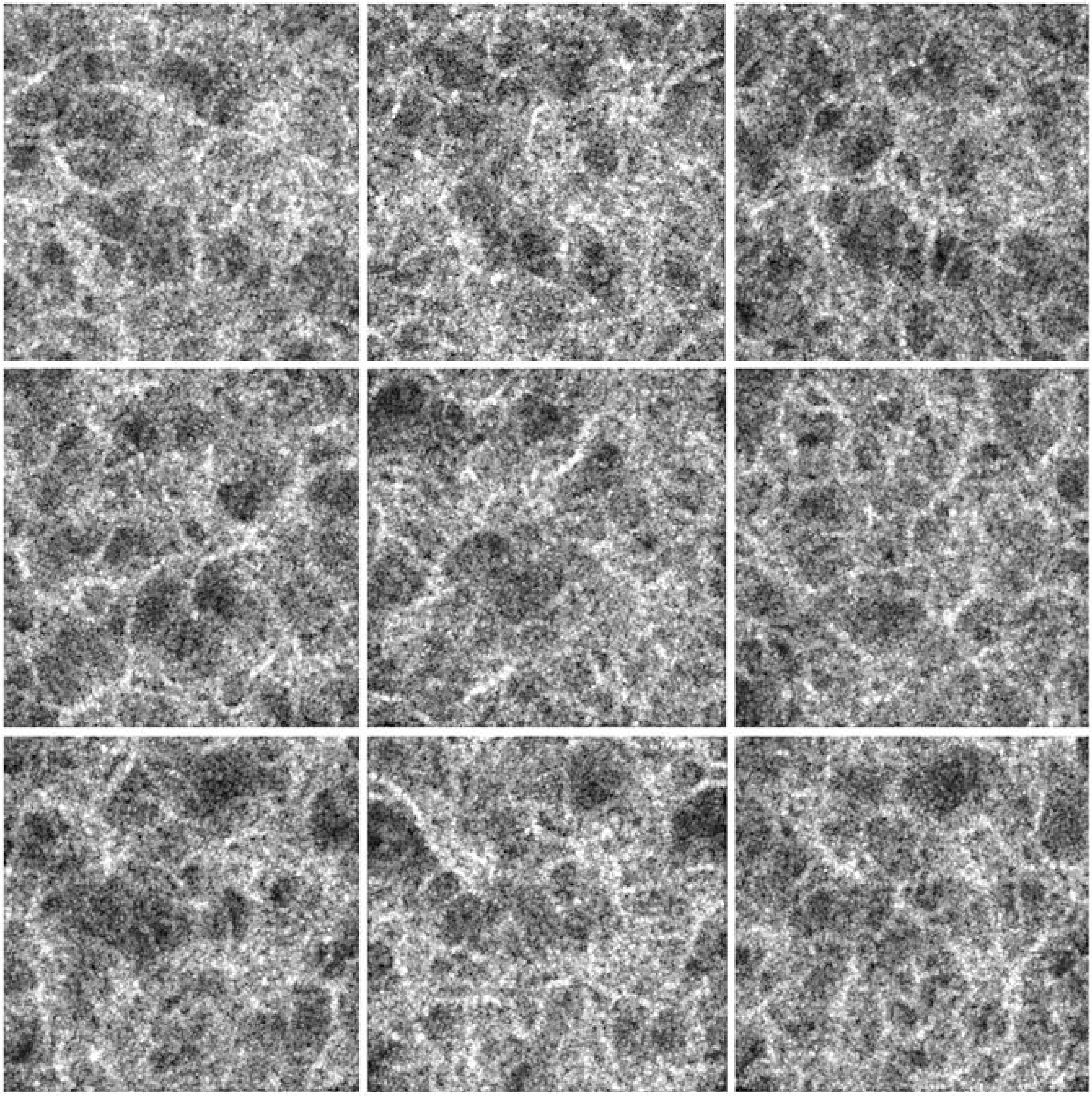

Figure S20. HRTEM images of the final configurations (at HIGH density – 1.15 g/cm^3, T = 2000 K) obtained after nine different massages, namely: MM1/MM4, MM1/MM6, MM1/MM8 (first row), MM2/MM4, MM2/MM6, MM2/MM8 (second row), MM3/MM4, MM3/MM6, MM3/MM8 (third row).

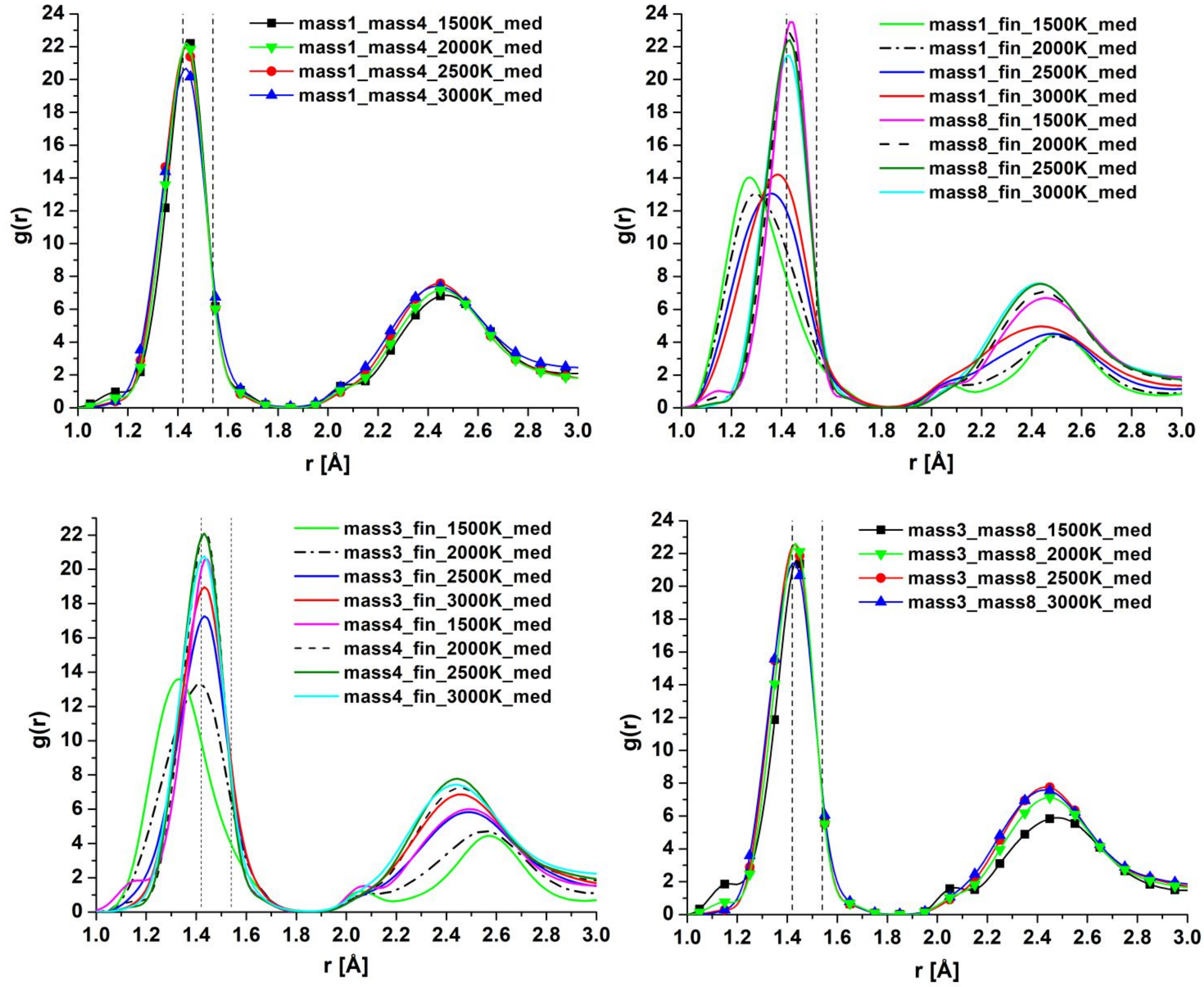

Figure S21. Plots of Pair Distribution Functions (PDF) or **g(r)** of the final configurations (at MEDIUM density – 0.50 g/cm^3) obtained after four different massages, namely: MM1/MM4, MM1/MM8, MM2/MM4, MM2/MM8 at different temperatures: T = 1500, 2000, 2500, 3000 K.

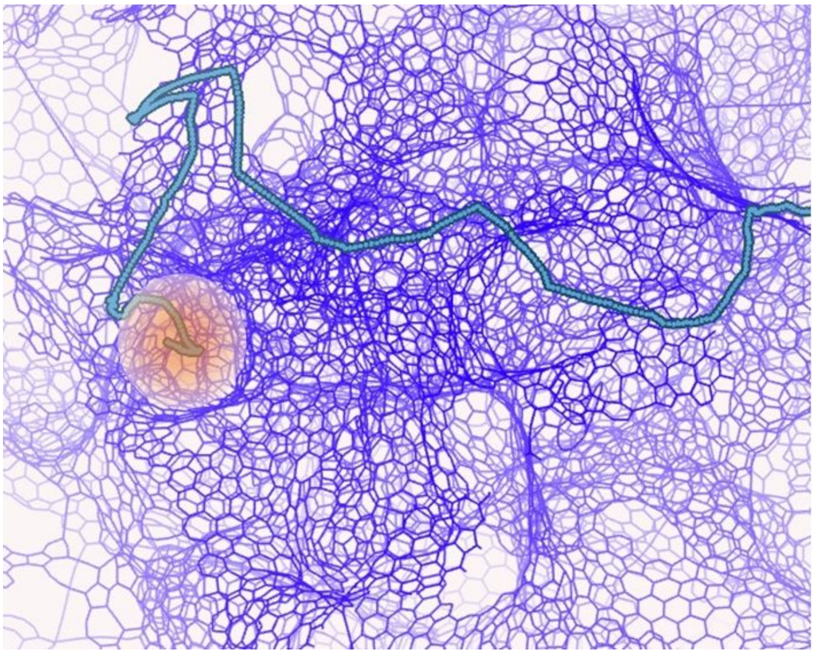

Figure S22. A narrow tunnel identified by the CAVER software in one of the sampled structures exhibiting a bimodal distribution of pore sizes. The tunnel corresponds to the continuous green line, and for the sake of clarity, we have highlighted with a pink aura its intersection with the wall of the periodic unit cell.

Bibliography

1 Plimpton, S. Fast Parallel Algorithms for Short-Range Molecular Dynamics. *J. Comput. Phys.* **1995**, *117*, 1–19

2 Berendsen, H.J.C.; Postma, J.P.M.; van Gunsteren, W.F.; DiNola, A.; Haak, J.R. Molecular Dynamics with Coupling to an External Bath. *J. Chem. Phys.* **1984**, *81*, 3684-3690

3 van Duin, A.C.T.; Strachan, A.; Stewman, S.; Zhang, Q.; Xu, X.; Goddard, W.A. $ReaxFF_{SiO}$ Reactive Force Field for Silicon and Silicon Oxide Systems. *J. Phys. Chem. A* 2003, 107, 3803-3811.

4 Nielson, K. D.; Van Duin, A. C. T.; Oxgaard, J.; Deng, W. Q.; Goddard, W. A. Development of the ReaxFF Reactive Force Field for Describing Transition Metal Catalyzed Reactions, with Application to the Initial Stages of the Catalytic Formation of Carbon Nanotu-bes. *J. Phys. Chem. A* **2005**, *109*, 493–499.

5 *ADF2019*, SCM, Theoretical Chemistry, Vrije Universiteit, Amsterdam, The Netherlands, 2019.
